# Structural insights into pterocarpan reductases unveil a universal ring-opening mechanism in plant biosynthesis of 4-(furan-2-yl) phenol derivatives

**DOI:** 10.1016/j.apsb.2026.01.005

**Published:** 2026-01-09

**Authors:** Hongye Li, Jianlin Zou, Meng Zhang, Chunxue Zhao, Yang-oujie Bao, Yanfang Yang, Min Ye

**Affiliations:** State Key Laboratory of Natural and Biomimetic Drugs, School of Pharmaceutical Sciences, Peking University, Beijing 100191, China

**Keywords:** Pterocarpan reductase, Catalytic mechanisms, Crystal structure, *Glycyrrhiza uralensis*, 4-(Furan-2-yl) phenol derivatives, Ancestral sequence reconstruction, C‒O bond cleavage

## Abstract

Pterocarpans and isoflavans are important phytoalexins, and demonstrate significant benefits to human health. Pterocarpan reductases (PTRs) catalyze the conversion of pterocarpans to isoflavans, while the catalytic mechanism remains unknown. Herein, we report six PTRs (GuPTR1–6) from *Glycyrrhiza uralensis*, together with the first PTR crystal structure (GuPTR1/(−)-medicarpin/NADP^+^, 1.8 Å). Structural analysis and mutagenesis reveal that a lysine-mediated deprotonation of the 7-OH group triggers C‒O bond cleavage of pterocarpans in the furan ring-opening reactions. This mechanism also applies to similar ring-opening enzymatic reactions. Through ancestral sequence reconstruction, we obtained a multifunctional reductase N0, which could accept different types of 4-(furan-2-yl) phenol derivatives as substrates. This study not only unveils the catalytic mechanisms of PTRs, but also provides a powerful enzymatic tool for the synthesis of bioactive isoflavans.

## Graphical abstract

This study reports the first crystal structure of pterocarpan reductase (GuPTR1/(−)-medicarpin/NADP^+^) and its catalytic mechanism. Ancestral sequence reconstruction yields a multifunctional reductase N0 for ring-opening reactions of 4-(furan-2-yl) phenol derivatives.

**Figure:**
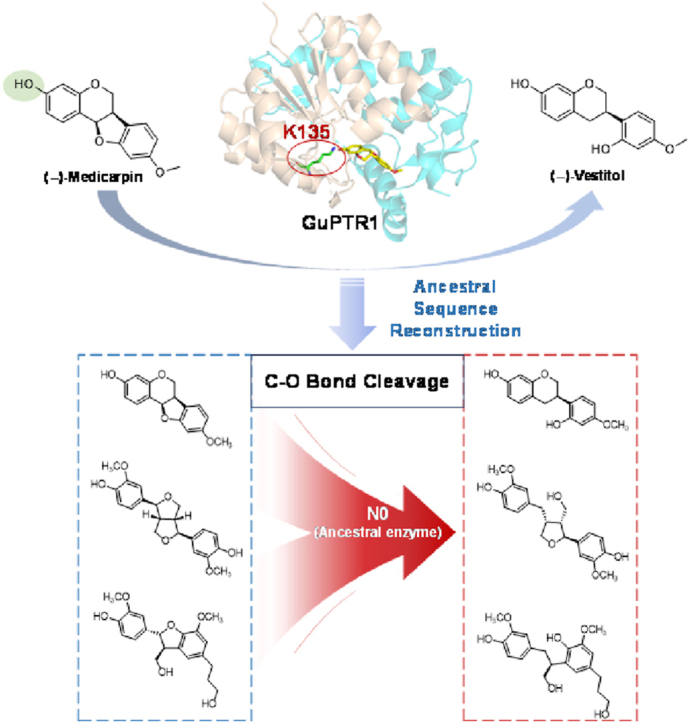

## 1 Introduction

Pterocarpans and isoflavans are ubiquitous secondary metabolites in leguminous plants, exhibiting remarkable pharmacological potential, including antitumor, skin-whitening, antibacterial, and antioxidant activities^1^, ^2^, ^3^, ^4^, ^5^. For instance, medicarpin from *Medicago alfalfa* can promote osteoblast differentiation and bone mineralization^6^, ^7^. The tyrosinase inhibitor glabridin from *Glycyrrhiza glabra* possesses skin-lightening capacity and is extensively employed in the market^8^, ^9^, ^10^, ^11^. Equol from *Glycine max* has promising applications in preventing cardiovascular disease, breast cancer, and prostate cancer due to its high antioxidant activity and hormone-like activity^12^. Licoricidin from *Glycyrrhiza uralensis* possesses anti-inflammatory, anticancer, and UV-induced photoaging inhibition properties^13^, ^14^, ^15^, ^16^, ^17^. On the other hand, these compounds serve as phytoalexins, playing a crucial role in plant defense responses against pathogenic microorganisms^18^. For example, medicarpin in *Medicago truncatula*^19^, ^20^, *Medicago alfalfa*^21^ and *Cicer arietinum*^22^, maackiain in *C. arietinum*^22^, pisatin in *Pisum sativum*^23^ as well as glyceollins I‒VI in soybean^24^ are related with resistance toward environmental stress. Therefore, the biosynthetic study of these compounds is valuable for their pharmaceutical and agricultural applications.

Pterocarpans were first identified as biosynthetic precursors of isoflavans through isotopic labeling experiments, where ^14^C-labeled medicarpin was fed to CuCl_2_-treated seedlings of *Medicago sativa*^25^. Akashi et al.^26^ identified the first pterocarpan reductases (PTRs), LjPTR1-4, from *Lotus japonicus*. These four enzymes could all convert medicarpin to vestitol. The authors also proposed a catalytic mechanism, in which an acidic amino acid residue acts as a proton donor, though no experimental evidences were provided. Elucidating the catalytic mechanisms of PTRs will promote the efficient synthesis of bioactive isoflavans, including glabridin^27^, licoricidin^13^, ^14^, ^15^, ^16^, ^17^, and equol^12^.

Aside from PTRs, phenylcoumaran benzylic ether reductase (PCBER) and pinoresinol-lariciresinol reductase (PLR) could also catalyze C‒O bond cleavage of 4-(furan-2-yl) phenol derivatives. PCBER catalyzes the reduction of phenylcoumaran benzylic ether lignans to their corresponding diphenols^28^. PLR catalyzes the reduction of pinoresinol to lariciresinol and then to secoisolariciresinol^29^. These enzymes are NADPH dependent, and belong to the short-chain dehydrogenase/reductase (SDR) family^26^^,^^30^. Phylogenetic analysis reveals that PTR and PCBER form a distinct clade, neighboring to the clade containing PLR. They represent two closely related subgroups evolved from a common SDR ancestor after a gene duplication event^26^.

In this study, we characterized six PTRs from the medicinal plant *Glycyrrhiza uralensis*, and obtained the crystal structure of GuPTR1 in complex with (−)-medicarpin and NADP^+^. Furthermore, we found that the ring-opening reaction catalyzed by PTRs was initiated by deprotonation of the 7-OH group through a highly conserved lysine residue. This mechanism is shared by PLR and PCBER, and facilitates the reconstruction of an ancestral enzyme N0 with a broad substrate scope.

## 2 Materials and methods

### 2.1 Materials and reagents

The fresh plants of *Glycyrrhiza uralensis* Fisch. (8–9 weeks) were collected from Inner Mongolia Autonomous Region of China for total RNA extraction and reverse transcription.

Compounds **1**–**2**, **17**–**18** and related catalyzed products were obtained from commercial sources (TargetMol, Shanghai, China; Desite, Chengdu, China). The other compounds for substrate screening were from the compound library of our laboratory. NADPH was purchased from Sigma‒Aldrich (St. Louis, MO, USA). HPLC analysis was performed on an Agilent 1260 instrument. LC/MS analysis was performed on a Q-Exactive quadrupole Orbitrap mass spectrometer (Thermo Scientific, San Jose, CA, USA) equipped with a heated electrospray ionization source (HESI). The LC/MS method parameters were listed in Supporting Information Table S1. To calculate the catalytic conversion rates, peak areas of both the substrate and product were integrated by Chromeleon® at a certaokin wavelength. Semi-preparative HPLC was performed on a Laballiance Series III instrument equipped with a Zorbax SB-C18 column (9.4 mm × 250 mm, 5 μm, Agilent, USA). The NMR spectra were recorded on a Bruker AVANCE III-400 instrument (Bruker, Karlsruhe, Germany) at 400 MHz for ^1^H and 100 MHz for ^13^C in DMSO-*d*_6_ using TMS as the reference.

### 2.2 Molecular cloning

The full-length candidate genes were amplified from cDNA with 2 × Hieff Canace Gold PCR Master Mix (YEASEN, China). Candidate genes of PTRs were recombined into the pET-28a (+) vector (Invitrogen, USA) at BamH I site. Sequences of the primers used in this study were listed in Supporting Information Tables S2 and S3.

### 2.3 Expression and purification of GuPTRs

The recombinant plasmids of PTRs were introduced into *Escherichia coli* BL21 (DE3) (Transgen Biotech, China) for heterologous expression. The *E. coli* cells were grown in 500 mL Luria-Bertani medium (JS0666, JSENB, China) containing kanamycin (50 μg/mL) at 37 °C. The cells were induced with 0.1 mmol/L IPTG at 18 °C when OD_600_ reached 0.4–0.6. After 18–24 h, the cell pellets were harvested by centrifugation (4000 rpm, 10 min at 4 °C), and then resuspended in 15 mL lysis buffer (50 mmol/L NaH_2_PO_4_ pH 8.0, 300 mmol/L NaCl, 30 mmol/L imidazole, pH 8.0). Then the cells were disrupted by sonication on ice, and the cell debris was removed by centrifugation at 7500 rpm for 50 min at 4 °C. The supernatant was collected and loaded onto a pre-equilibrated column (His Trap™ HP, 5 mL, GE Healthcare), and eluted with different concentrations of elution buffer (50 mmol/L NaH_2_PO_4_, pH 8.0, 300 mmol/L NaCl, 30 or 300 mmol/L imidazole)^31^.

### 2.4 Enzyme activity assay

The purified proteins were used for functional characterization by *in vitro* enzymatic reactions. The reactions were conducted in 100 μL NaH_2_PO_4_‒Na_2_HPO_4_ buffer (50 mmol/L, pH 7.4) containing 50 μg purified enzymes. The incubation mixtures include substrates **1**–**18** (0.1 mmol/L) and nicotinamide adenine dinucleotide phosphate (NADPH, 0.5 mmol/L). The reactions continued in a shaking incubator for 1 h (30 °C). All reactions were terminated by adding 100 μL ice-cold methanol. The mixtures were then centrifuged at 15,000 rpm for 30 min. The supernatants were analysed by HPLC and LC/MS.

### 2.5 Crystallization and structural determination

The full-length cDNA of GuPTR1 was cloned into pET-28a (+) vector. The S-tag of pET28a was removed. A TrxA-tag and 6 × His-tag followed by thrombin site were added before the N-terminus of the target protein to facilitate purification. The TrxA-His-thrombin-GuPTR1 protein was expressed in *E. coli* BL21(DE3) strain and purified by Ni-affinity chromatography (GE Healthcare). After purification, the recombinant protein was digested by thrombin to remove the tag (4 °C, 10 h). The protein solution after removal of His-tag was purified by Ni-NTA column again. Subsequently, the fraction was further purified using a size-exclusion chromatography on a Superdex™ 200 increase 10/300 GL prepacked column (GE Healthcare). The elution buffer was 20 mmol/L Tris-HCl (pH 7.5) and 50 mmol/L NaCl. The purity of target protein was 95%. The protein concentration was determined by the B-500 Biophotometer (METASH, China). The purified GuPTR1 was concentrated to 20 mg/mL.

Purified GuPTR1 was incubated with 5 mmol/L NADP^+^ and 5 mmol/L (−)-medicarpin (**1**) at 4 °C for 2 h before setting up the crystallization trays. The crystals were prepared by hanging drop vapor diffusion. The condition of crystal growth: 0.05 mol/L sodium sulfate, 0.05 mol/L lithium chloride, 0.05 mol/L Tris-HCl pH 8.5, 32% *v*/*v* PEG 400, 16 °C, 7 days. The crystals were flash-frozen in the reservoir solution supplemented with 25% (*w*/*v*) ethylene glycol.

The diffraction data of GuPTR1 crystals were collected at beamlines BL19U1 and BL02U1 Shanghai Synchrotron Radiation Facility (SSRF). The data were processed with XDS. The structures were solved by molecular replacement with Phaser. Crystallographic refinement was performed repeatedly with Phenix and COOT. The refined structures were validated by Phenix and the PDB validation server (https://validate-rcsb-1.wwpdb.org/). The final refined GuPTR1/NADP^+^/(−)-medicarpin structure was deposited in Protein Data Bank with the access ID 9UM2.

### 2.6 Site-directed mutagenesis of GuPTR1 and enzyme activity assay

GuPTR1 mutants (G116A, I117A, I117P, L131A, F132A, K135A, K135C, K135D, K135E, K135F, K135G, K135H, K135I, K135L, K135M, K135N, K135P, K135Q, K135R, K135S, K135T, K135V, K135W and K135Y) were constructed using 2 × Hieff Canace Gold PCR Master Mix (YEASEN, China) according to the manufacturer's instructions. The corresponding primers designed to construct the site-directed mutants are listed in Table S3. After verification of the mutant sequences, the recombinant plasmids were transformed into *E. coli* BL21(DE3) for heterologous expression. The protein expression, purification and enzyme activity assays of mutants were performed under the same conditions.

### 2.7 Determination of kinetic parameters of GuPTR1, GuPTR1_K135A_, and GuPTR1_I117P_

Kinetic parameters of GuPTR1 reactions were calculated using (−)-medicarpin (**1**) as substrate. Assays were performed in a final volume of 100 μL, consisting of 50 mmol/L Na_2_HPO_4_‒NaH_2_PO_4_ (pH 7.4), 0.5 ng GuPTR1, 10 mmol/L of saturated NADPH, and different concentrations of **1** (0.01, 0.025, 0.05, 0.1, 0.25, 0.5, 1, 1.5, 2 μmol/L). After incubating at 30 °C for 10 min, the reactions were quenched with ice cold MeOH and centrifuged at 15,000 rpm for 20 min, and the supernatants were analyzed by HPLC. Samples were separated on a Zorbax SB-C18 column (4.6 mm × 75 mm, 3.5 μm, Agilent, USA) protected with a Zorbax Extend C18 guard column (4.6 mm × 12.5 mm, 5 μm). The mobile phase consisted of methanol (B) and water containing 0.1% (*v*/*v*) formic acid (A). A linear gradient elution program was used: 0 min, 40% B; 3 min, 70% B; 6 min, 100% B. All experiments were performed in triplicate (*n* = 3). Unless otherwise stated, the kinetic parameters in this work were calculated using the method of Michaelis‒Menten plot.

Kinetic parameters of GuPTR1_K135A_ reaction were calculated using **1** as substrate. Assays were performed in a final volume of 100 μL, consisting of 50 mmol/L Na_2_HPO_4_‒NaH_2_PO_4_ (pH 7.4), 13.3 ng GuPTR1_K135A_, 10 mmol/L of saturated NADPH, and different concentrations of **1** (0.5, 1, 2, 4, 6, 8, 10, 15, 20, 40, 60, 80, 100, and 200 μmol/L). After incubating at 30 °C for 10 min, the reactions were quenched with ice-cold MeOH and centrifuged at 15,000 rpm for 20 min, and the supernatants were analyzed by HPLC. The chemical analysis of samples was conducted following the same method as mentioned above. The kinetic parameters were calculated using the method of Michaelis‒Menten plot.

Kinetic parameters of GuPTR1_I117P_ reaction were calculated using **1** as substrate. Assays were performed following the same method as GuPTR1_K135A_.

Kinetic parameters of N0 toward **1** were calculated as follows. Assays were performed in a final volume of 100 μL, consisting of 50 mmol/L Tris-HCl (pH 7.0), 1.25 ng N0, 10 mmol/L of saturated NADPH, and different concentrations of **1** (1, 2.5, 5, 10, 20, 40, 60, 80, 100, 150, and 200 μmol/L). After incubating at 60 °C for 10 min, the reactions were quenched with ice cold MeOH and centrifuged at 15,000 rpm for 20 min, and the supernatants were analyzed by HPLC. Chemical analysis of samples was conducted following the same method as mentioned above.

Kinetic parameters of N0 toward (+)-pinoresinol (**17**) were calculated as follows. Assays were performed in a final volume of 100 μL, consisting of 50 mmol/L Tris-HCl (pH 7.0), 2.00 ng N0, 10 mmol/L of saturated NADPH, and different concentrations of **17** (2, 4, 6, 8, 10, 20, 40, 60, 80, and 100 μmol/L). Assays were performed following the same method as N0 toward **1**.

Kinetic parameters of N0 toward (−)-(2*R*,3*S*)-dihydrodehydrodiconiferyl alcohol (**18**) were calculated as follows. Assays were performed in a final volume of 100 μL, consisting of 50 mmol/L Tris-HCl (pH 7.0), 7.14 ng N0, 10 mmol/L of saturated NADPH, and different concentrations of **18** (1, 2.5, 5, 10, 20, 40, 60, 80, 100, 200, 400, 800, 1000, and 2000 μmol/L). Assays were performed following the same method as N0 toward **1**.

### 2.8 Molecular dynamics simulation of GuPTR1 and GuPTR1_I117P_

Molecular dynamics (MD) simulations were performed using the Amber16 software package^32^. The Amber ff14SB force field was applied for the protein^33^. Force field parameters of the ligands were generated using the antechamber module of Amber16 and the general Amber force field (GAFF)^34^. The structure was immersed into a truncated octahedral box that extended 8 Å away from the solute border, using the TIP3P water model and periodic boundary conditions^35^. Seven Na^+^ ions were added into the box to neutralize the system. The water and ions were initially minimized for 5000 steps using the steepest descent method for the first 3000 steps and then the conjugate gradient algorithm for the last 2000 steps, with the position of protein and ligands fixed (force constant was 500 kcal/(mol⋅Å^2^)). In the second energy minimization stage, the restraints on protein and ligands were removed. This stage was conducted for 10,000 steps, using the steepest descent method in the first 5000 steps and then the conjugate gradient algorithm for the last 5000 steps. After that, a heat-up MD was run at a constant volume. The system was heated from 0 to 300 K for 100 ps with a weak restraint of 10 kcal/(mol⋅Å^2^) on the solute. Then, free MD simulations of 100 ns were carried out under the NPT condition utilizing the GPU accelerated pmemd. cuda code. All the MD results were analyzed using the ptraj module of the Amber16 software package^32^.

### 2.9 Scaled-up reactions

To prepare the catalytic product of **3**, the reaction mixtures contained 10 mL buffer (10 mmol/L PBS, pH 7.4), 0.2 mmol/L **3** (7.10 mg dissolved in DMSO), 1.0 mmol/L NADPH and 300 μg purified protein of GuPTR1. A total of 25 parallel tube reactions were conducted. The reactions were performed at 30 °C overnight and terminated by extraction with a 4-fold volume of ethyl acetate. The organic solvent was removed under reduced pressure. The residue was dissolved in 5.0 mL of methanol. The products were then purified by reversed-phase semi-preparative HPLC. The pure product (6.20 mg) was dissolved in DMSO-*d*_6_ to record the NMR spectra. The structures were characterized by HRMS and extensive 1D and 2D NMR analyses.

### 2.10 Phylogenetic analyses and ancestral sequence reconstruction

Amino acid sequences of PTRs, PLRs, PCBERs in the CNGB database were acquired by BLASTP in the 1000 Plants Project Database. The phylogenetic tree was constructed using MEGA 11 Software with the maximum likelihood method based on ClustalW multiple alignments^36^. The ancestral sequence of these enzymes was reconstructed by GRASP according to the sequence alignment result and the phylogenetic tree^37^.

## 3 Results and discussion

### 3.1 GuPTR1–6 could catalyze (−)-medicarpin to (−)-vestitol

**Figure 1:**
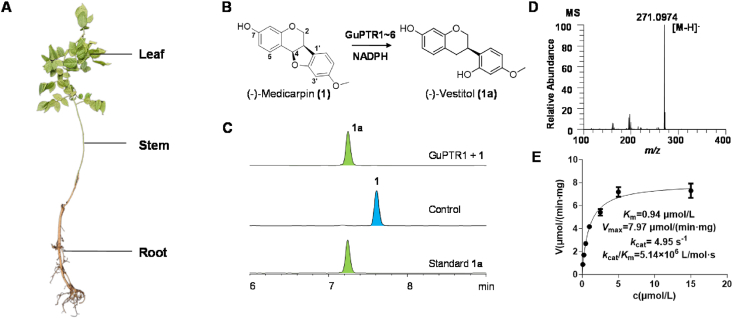
Functional characterization of GuPTR1. (A) A picture of *Glycyrrhiza uralensis*; (B) The reaction catalyzed by GuPTR1–6; (C) HPLC analysis of the reaction mixture of GuPTR1 (*λ* = 280 nm); (D) The (−)-ESI-MS spectrum of **1a**; (E) The kinetic parameters of GuPTR1 with **1** as substrate. Three biologically independent samples were tested (*n* = 3).

To identify potential PTR genes in *G. uralensis* (Fig. 1A), we performed a local blastn search using *LjPTR1–4* sequences (Supporting Information Table S4) as templates against the *G. uralensis* transcriptome^38^. Six candidate PTR genes (GuPTR1–6) were acquired with *e*-value <10^−20^ (Supporting Information Table S5). After sub-cloning these genes into pET-28a (+) vectors and expressing them in *Escherichia coli*, we characterized their functions through *in vitro* enzymatic reactions. HPLC analysis revealed that incubation of GuPTR1–6 proteins with (−)-medicarpin (**1**) and NADPH generated a new product (Fig. 1B and C, Supporting Information Fig. S1). The product exhibited a [M‒H]^‒^ ion at *m*/*z* 271.0974 in the mass spectrum, which was 2 Da greater than (−)-medicarpin (Fig. 1D). By comparing with a reference standard, we identified this product as (−)-vestitol (**1a**). Among GuPTR1–6, GuPTR1 demonstrated the highest catalytic efficiency (*k*_cat_/*K*_m_ = 5.14 × 10^6^ L/mol·s, Fig. 1E), and was more potent than the previously reported PTRs^26^.

### 3.2 GuPTR1–6 only recognize pterocarpans with 7-OH as substrates

**Figure 2:**
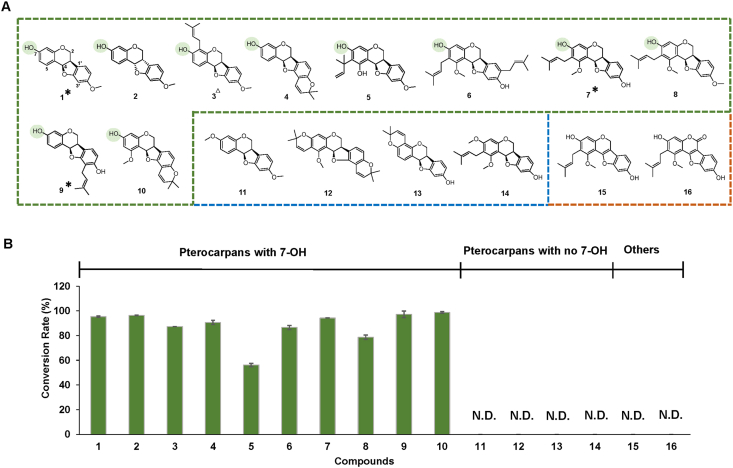
Substrate promiscuity of GuPTR1. (A) Structures of substrates **1**–**16**; (B) The conversion rates (%) of GuPTR1 towards **1**–**16**. Data are presented as mean ± SD, *n* = 3 (three biologically independent samples were tested). ∗, the products were confirmed by comparing with reference standards; ^Δ^, the product was purified in this work and identified by NMR analysis. N.D., no products were detected.

Next, we tested the substrate promiscuity of GuPTR1–6 with 16 compounds (**1**–**16**) (Fig. 2A). According to LC/MS analysis, GuPTR1 could catalyze all the pterocarpans with 7-OH, but no pterocarpans without 7-OH (**11**–**14**) or those with a double bond between C-3 and C-4 (**15**, **16**) (Fig. 2B, Supporting Information Figs. S1–S16). Although 7-OH is spatially distant from the furan ring-opening site, it unexpectedly influences PTR-catalyzed conversion. The enzymatic products of compounds **1**, **7**, and **9** were identified as (−)-vestitol (**1a**), glyasperin C (**7a**), and phaseollidin isoflavan (**9a**), respectively, by comparing with reference standards^39^. The product of compound **3** was purified from a scaled-up enzymatic reaction, and its structure was identified by NMR spectroscopic analysis (Supporting Information Figs. S17–S20). The two hydrogen signals of H-4 at *δ*_H_ 2.70 and *δ*_H_ 2.89 indicated cleavage of the C‒O bond at the furan ring (Supporting Information Table S6). Among the other GuPTRs, GuPTR6 displayed catalytic functions almost identical to that of GuPTR1, while GuPTR2/3/4/5 could not catalyze most prenylated substrates (Supporting Information Table S7).

### 3.3 The lysine-medidated deprotonation of 7-OH initiates the catalysis of GuPTR1

**Figure 3:**
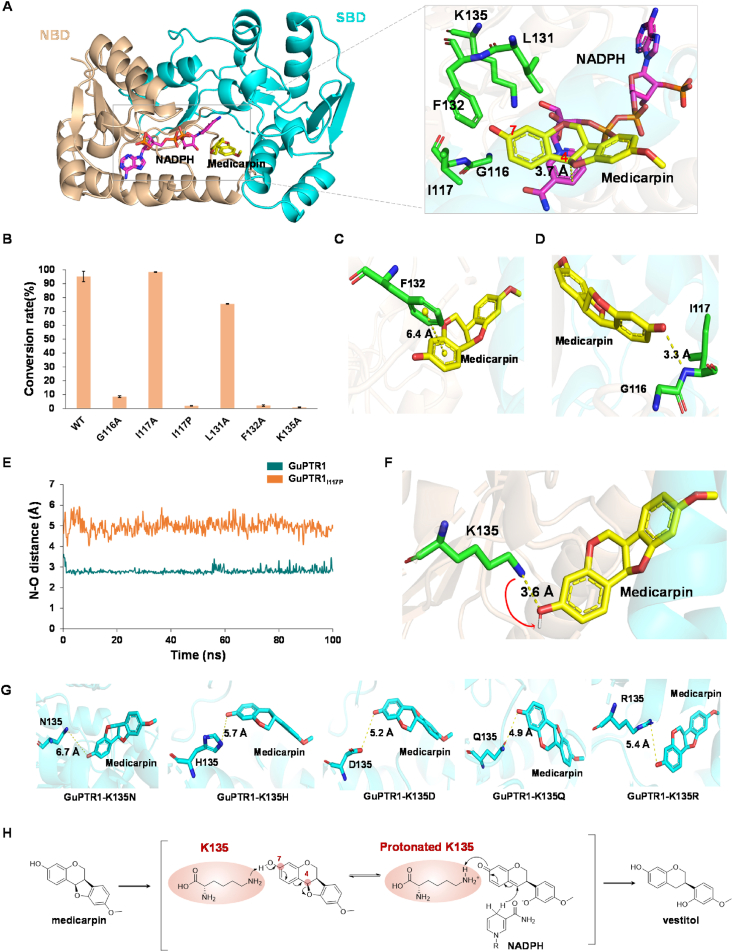
The catalytic mechanisms of GuPTR1. (A) The crystal structure of GuPTR1 in complex with (−)-medicarpin and NADP^+^. (B) The conversion rates of GuPTR1 and its mutants (%). Data are presented as mean ± SD (*n* = 3, three independent samples were tested). WT, wild type. (C) Interactions between F132 and (−)-medicarpin. (D) Interactions between G116, I117 and (−)-medicarpin. (E) Calculated distance between the N atom on the side chain of K135 and the *O* atom of 7-OH of (−)-medicarpin in 100 ns MD simulations of GuPTR1_I117P_ mutant and GuPTR1 (*n* = 3, data were obtained from three independent replicates). (F) Interactions between K135 and (−)-medicarpin. (G) Binding modes of (−)-medicarpin with K135 mutants of GuPTR1. (H) A proposed catalytic mechanism of GuPTR1.

To interpret the catalytic mechanisms of PTRs, we cultured protein crystals and obtained the crystal structure of GuPTR1 in complex with (−)-medicarpin and NADP^+^ through X-ray diffraction (1.8 Å, PDB: 9UM2) (Supporting Information Fig. S21, Table S8), which represents the first crystal structure of PTR enzymes. The structure of GuPTR1 contains two domains, with the NADPH binding domain (NBD) on the N-terminal and the substrate binding domain (SBD) on the C-terminal. The NBD consists of seven *β*-strands surrounded by six *α*-helices, and the SBD contains two *β*-strands and five *α*-helices. (−)-Medicarpin and NADP^+^ are located within the groove between NBD and SBD (Fig. 3A). The distance between the reaction center of (−)-medicarpin (C-4) and NADPH is 3.7 Å, indicating that NADPH may serve as a hydride donor in the catalysis of PTRs, similar to its function in other SDRs^40^.

To illustrate the role of 7-OH in the catalytic reaction, we performed an alanine scanning of amino acid residues within 5 Å of 7-OH of (−)-medicarpin in the crystal structure (Fig. 3A). Among the five mutants, the catalytic activities of G116A, F132A and K135A decreased significantly, indicating their significance in the catalysis of PTRs despite their long distance from the furan ring (Fig. 3B, Supporting Information Table S9). Molecular interaction analysis using PLIP 2021^41^ demonstrated that the benzene ring of F132 could form a T-shaped *π*-stacking with the A ring of (−)-medicarpin, with a distance of 6.4 Å between the two phenyl ring centroids, within the preferential distance between 4.5 and 7.0 Å^42^ (Fig. 3C). The small size of G116 allows 7-OH of (−)-medicarpin to be placed nearby, and the amide imino group of I117 could form a hydrogen bond with 7-OH. These interactions stabilize the substrate during the catalysis process (Fig. 3D). While mutagenesis of I117 to alanine hardly affected the activity, we mutated I117 to proline, an imino acid (Supporting Information Fig. S22). Given that no imino hydrogen is present to form hydrogen bonding with 7-OH, the I117P mutant showed remarkably decreased catalytic activity, with *k*_cat_/*K*_m_ of 335.29 L/mol·s (Fig. 3B, Supporting Information Fig. S23, Table S9). Thus, F132, G116, and I117 of GuPTR1 play a key role in stabilizing the substrate.

The K135A mutant showed even weaker activity than the above mutants, with *k*_cat_/*K*_m_ value of 117.82 L/mol·s (Fig. 3B, Fig. S23, Table S9). This result, together with the previously reported hypothesis^26^ (Supporting Information Fig. S24), indicates that K135 may initiate the deprotonation of 7-OH, trigger electron redistribution, and ultimately lead to cleavage of the C‒O bond. To further verify the role of K135, we conducted molecular dynamics (MD) simulations of GuPTR1 and GuPTR1_I117P_ using Amber16^32^. The protein structures were proven reliable according to the stable states within 100 ns (Supporting Information Fig. S25). Specifically, we calculated the distance between the N atom on the side chain of K135 and O-7 of (−)-medicarpin in both GuPTR1 and GuPTR1_I117P_. The long N‒O distance in GuPTR1_I117P_ renders the deprotonation of 7-OH difficult. This result was consistent with the decreased activity of the I117P mutant, though this mutagenesis did not affect the distance between C-4 and NADPH (Fig. 3E, Supporting Information Figs. S26 and S27). Furthermore, a site-directed saturation mutagenesis screening also supported the significance of K135, as all the mutants showed very weak activities (Supporting Information Fig. S28, Table S9). We modelled the structures of five representative mutants K135N, K135H, K135D, K135Q and K135R using the crystal structure of GuPTR1 as template *via* SWISS-MODEL^43^. All the simulated structures showed longer N‒O distance (4.9‒6.7 Å) than the wild type protein (3.6 Å) (Fig. 3F and G). The long side chain of lysine leads to a short N‒O distance and facilitates the deprotonation of 7-OH. The above results confirmed the significance of K135 in the deprotonation of 7-OH to initiate the ring-opening reaction.

Based on the above results, we proposed the catalytic mechanisms of GuPTR1. The reaction is initiated by K135 to abstract the proton from 7-OH of medicarpin. This deprotonation generates a negatively charged oxyanion intermediate, where the free electron is delocalized across the adjacent conjugated *π*-system. Concurrently, electron density redistrubution activates the system to facilitate subsequent C‒O bond cleavage and the formation of a quinoid intermediate. Then, C-4 of medicarpin accepts a hydride from NADPH, leading to the restoration of aromaticity. Finally, the protonated lysine donates the proton back to O-7, regenerating a hydroxyl group (Fig. 3H).

### 3.4 The key lysine residue is highly conserved in plant PTRs

**Figure 4:**
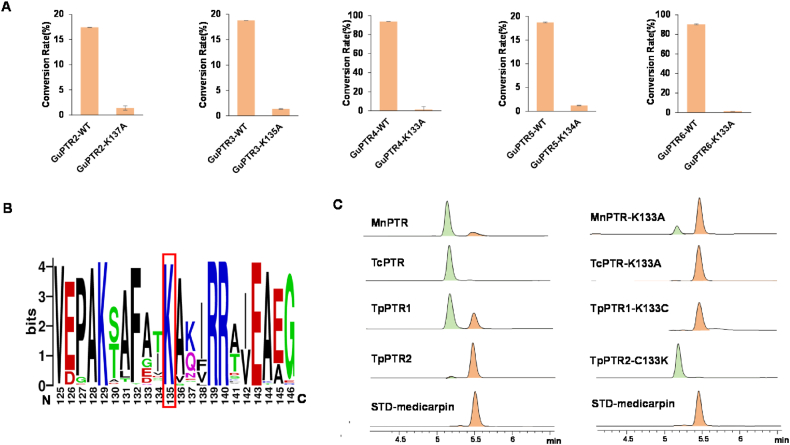
Significance of the lysine residue in the catalytic reaction of PTR enzymes. (A) Conversion rates (%) of GuPTR2-6 and their single-site mutants catalyzing (−)-medicarpin into (−)-vestitol. Data are presented as mean ± SD (*n* = 3, three independent samples were tested); (B) Partial amino acid sequence alignment of 100 potential PTRs from CNGB; (C) HPLC chromatograms for the enzymatic reactions catalyzed by MnPTR, TcPTR, TpPTR1 and TpPTR2, and their single-site mutants (*λ* = 280 nm). WT, Wild type.

To investigate the conservatism of the key lysine in PTRs, we mutated the lysine in GuPTR2–6 to alanine. The catalytic activities decreased markedly, demonstrating the significance of the conserved lysine residue (Fig. 4A, Supporting Information Table S10). We further screened 100 potential plant PTRs in CNGB database through a BLASTP project^44^. A sequence alignment indicated the key lysine residue was highly conserved among all the PTRs (Fig. 4B). It could be found in 99 PTRs except for *TpPTR2* from *Tiarella polyphalla*. We randomly selected three PTR genes (*TpPTR1*, *MnPTR*, *TcPTR*) containing the lysine residue from *Tiarella polyphalla*, *Morus nigra*, and *Tamarix chinensis*, respectively, along with *TpPTR2* which contains a cysteine instead of lysine. *In vitro* functional assays demonstrated that all the three lysine-containing PTRs (TpPTR1, MnPTR, TcPTR) could efficiently catalyze the conversion of (−)-medicarpin to (−)-vestitol, whereas TpPTR2 showed very weak catalytic activity despite its high sequence identity with TpPTR1 (Fig. 4C, Supporting Information Table S11). Site-directed mutagenesis of the conserved catalytic lysine to alanine in both MnPTR and TcPTR resulted in remarkable decrease of activity. The complementary mutation of TpPTR1 and TpPTR2 also resulted in expected activity changes (Fig. 4C, Table S11). The high conservation of the critical lysine residue in plant PTRs underscores its critical role in the catalytic activity of this enzyme family.

### 3.5 Reconstruction of a multifunctional ancestral enzyme N0

Similar to the above PTRs, pinoresinol-lariciresinol reductases (PLRs) and phenylcoumaran benzylic ether reductases (PCBERs) from plants could also catalyze ring-opening reactions of compounds with a 4-(furan-2-yl) phenol substructure. Through amino acid sequence alignment of reported PTRs^26^, PLRs^30^^,^^45^, ^46^, ^47^, ^48^, PCBERs^30^^,^^45^, and GuPTR1–6, we found that K135 of GuPTR1 was highly conserved among these enzymes, indicating they may share a similar catalytic mechanism (Supporting Information Fig. S29). In 2003, Min et al.^45^ determined the crystal structure of TpPLR1 and found the K138A mutation remarkably reduced its catalytic activity. They proposed that lysine served as a critical amino acid in PLR-catalyzed reactions. However, Xiao et al.^48^ analyzed the structure of IiPLR1 and proposed that the lysine residue was positioned too far away from the ring-opening site to directly participate in the catalysis. Instead, they suggested that it may play a role in stabilizing NADPH. By comparing the structures of GuPTR1 and IiPLR1, we found that both enzymes contain the NADPH binding domain (NBD) on the N-terminal and substrate binding domain (SBD) on the C-terminal (Supporting Information Fig. S30A). The locations of NADPH and substrates in the structures were similar, and the distance between the amine of K144 in IiPLR1 and 4-OH of (+)-pinoresinol (3.8 Å) was similar to the distance between K135 in GuPTR1 and 7-OH of medicarpin (3.6 Å) (Fig. S30B). Therefore, we speculate that similar catalytic mechanisms of GuPTR1 exist in PLR and PCBER (Supporting Information Fig. S31). The lysine residue deprotonates 4-OH and initiates the electron transfer chain. With the participation of NADPH, the reaction proceeds through a quinoid intermediate, ultimately yielding the corresponding ring-opened product.

**Figure 5:**
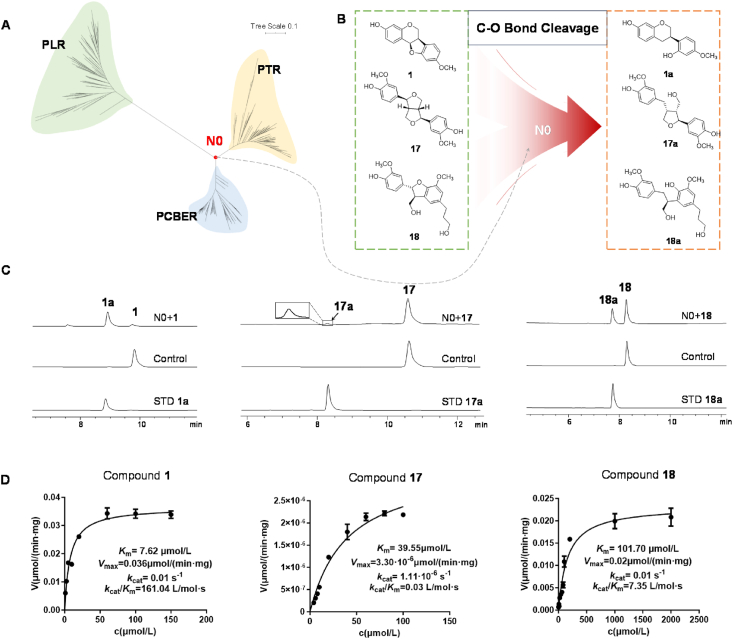
Reconstruction and catalytic functions of N0. (A) Phylogenetic tree analysis of plant PTRs, PLRs, and PCBERs; (B) Reactions catalyzed by N0; (C) HPLC chromatograms of N0 catalyzed reactions with **1**, **17** and **18** as substrates (*λ* = 280 nm); (D) Kinetic parameters of N0 with **1, 17** and **18** as substrates. Three independent samples were tested (*n* = 3).

While PLRs, PTRs, and PCBERs share similar catalytic mechanisms, they recognize different types of substrates^45^, ^46^, ^47^, ^48^ (Supporting Information Figs. S32 and S33). Therefore, we tried to construct a multifunctional ancestral enzyme which could accept all the three types of substrates. Using reported sequences as templates, we conducted a BLASTP search in the CNGB database^36^, identifying 100 potential PLRs and 100 PCBERs. PTRs and PLRs are widely distributed in angiosperms, present in 40 and 59 families, respectively. In contrast, PCBERs are exclusively present in bryophytes and gymnosperms, indicating their ancestral origin in plant evolution (Supporting Information Fig. S34). To explore their evolutionary relationships, we constructed a maximum likelihood (ML) tree incorporating all the 300 reductases (100 PTRs, 100 PLRs, and 100 PCBERs). Although PTRs and PLRs both co-occur in angiosperms, they form distinct evolutionary clades. Interestingly, PTRs exhibit a closer phylogenetic relationship with PCBERs (Fig. 5A), implying that PCBERs evolve to PTRs, whereas PLRs evolve independently.

To develop a general biocatalyst for these ring-opening reactions, we constructed the common ancestral sequence (N0) of PTR, PLR and PCBER using GRASP^36^. Subsequently, N0 was expressed and purified, and its activities were tested against substrates (−)-medicarpin (**1**), (+)-pinoresinol (**17**), and (−)-(2*R*,3*S*)-dihydrodehydrodiconiferyl alcohol (**18**). Intriguingly, N0 demonstrated catalytic activity towards all the three substrates (Fig. 5B and C). It showed the highest catalytic efficiency at 60 °C in Tris-HCl buffer (pH 7.0) (Supporting Information Fig. S35). The *k*_cat_/*K*_m_ value of N0 catalyzing substrate **1**, **17** and **18** was 161.04, 0.03 and 7.35 L/mol·s respectively (Fig. 5D). For substrate **18**, N0 was more efficient than the reported PCBERs^29^.

## 4 Conclusions

In this work, we characterized six pterocarpan reductases (GuPTR1–6) from *G. uralensis*, and solved the first PTR crystal structure (GuPTR1 in complex with (−)-medicarpin/NADP^+^). We further unraveled the catalytic mechanisms of PTRs, where a conserved lysine residue initiates deprotonation of pterocarpan 7-OH, and triggers the C‒O bond cleavage to form isoflavans. This catalytic mechanism is shared by related reductases including PLRs and PCBERs, despite their divergent substrate specificities. We reconstructed an ancestral enzyme N0, which could catalyze the ring-opening reactions of different types of 4-(furan-2-yl) phenol derivatives. This work unveils the ring-opening reaction mechanisms of plant PTRs, which are involved in the biosynthesis of a series of natural products with significant pharmacological potential.

## Author contributions

Min Ye for conceptualization; Hongye Li and Jianlin Zou for major investigation; Meng Zhang, Chunxue Zhao, Yangoujie Bao and Yanfang Yang assisted with data acquisition; Min Ye and Jianlin Zou for funding acquisition; Hongye Li and Jianlin Zou for writing-original draft; Min Ye for writing-review & editing.

## Conflicts of interest

The authors declare no conflicts of interest.

## Appendix A Supporting information

The following is the Supporting Information to this article.
