# Supplementary material for: Structural insights into pterocarpan reductases unveil a universal ring-opening mechanism in plant biosynthesis of 4-(furan-2-yl) phenol derivatives
Source: Acta Pharm Sin B. 2026 Jan 9;16(6):3953–62. doi: 10.1016/j.apsb.2026.01.005 (PMC13304732; doi:10.1016/j.apsb.2026.01.005)
Supplement: Multimedia component 1 [file mmc1.pdf]

Supporting Information for

Original article

Structural insights into pterocarpan reductases unveil a universal ring-opening mechanism in plant biosynthesis of 4-(furan-2-yl) phenol derivatives

Hongye Li†, Jianlin Zou†, Meng Zhang, Chunxue Zhao, Yang-oujie Bao, Yanfang Yang, Min Ye*

State Key Laboratory of Natural and Biomimetic Drugs, School of Pharmaceutical Sciences, Peking University, Beijing 100191, China

Received 28 July 2025; received in revised form 31 October 2025; accepted 10 November 2025

*Corresponding author.

E-mail address: yemin@bjmu.edu.cn (Min Ye).

†These authors made equal contributions to this work.

Contents

Tables

Table S1. HPLC and LC/MS methods used in this study.

Table S2. Primers for candidate genes of PTR.

Table S3. Primers for GuPTR1 mutants.

Table S4. PTR genes for BLAST analysis.

Table S5. GenBase accession numbers of GuPTR1–6.

Table S6. Assignment of NMR signals of **3a**.

Table S7. Catalytic activities of GuPTR1–6 utilizing **1–16**.

Table S8. Data collection, and refinement statistics of crystal structure.

Table S9. Catalytic activities of GuPTR1-mutants utilizing **1**.

Table S10. Catalytic activities of GuPTR2–6 and related K–A mutants utilizing **1**.

Table S11. Catalytic activities of MnPTR, TcPTR, TpPTR1, TpPTR2 and their mutants utilizing **1.**

Figures

Figures S1–S16. Catalytic activities of GuPTR1–6 toward **1–16**.

Figures S17–S20. NMR spectra of reduced product **3a**.

Figure S21. Crystal picture of GuPTR1.

Figure S22. Interactions between the three amino acid residues at position 117 and medicarpin.

Figure S23. Kinetic analysis for $GuPTR1_{K135A}$ and $GuPTR1_{I117P}$ against medicarpin.

Figure S24. Putative reaction scheme of PTR in previous work.

Figure S25. RMSD of protein structures of GuPTR1 and $GuPTR1_{I117P}$ within 100 ns.

Figure S26. Three independent molecular dynamics of N–O distance of $GuPTR1_{I117P}$ mutant and GuPTR1 in 100 ns.

Figure S27. Distance between the C-4 atom on the substrate and the hydride-donating site of NADPH in 100 ns MD simulation of $GuPTR1_{I117P}$ mutant and GuPTR1.

Figure S28. Site-saturation mutagenesis screening of K135 (*n*=3).

Figure S29. Sequence alignment of PTRs with PCBERs and PLRs.

Figure S30. Structural alignment of GuPTR1 and IiPLR1.

Figure S31. Putative catalysis mechanism of PLR and PCBER.

Figure S32. Catalytic activities of GuPTR1–6 toward **17**.

Figure S33. Catalytic activities of GuPTR1–6 toward **18**.

Figure S34. Distribution of PTR, PLR and PCBER in the plant kingdom.

Figure S35. Biochemical properties of N0.

References

Table S1. HPLC and LC/MS methods used in this study.

Method	Instrument	HPLC column	Mobile phase gradient
A	Agilent HPLC 1260	Zorbax SB-C18 column (4.6 mm×75 mm, 3.5 μm, Agilent, USA)	0 min 40% B;<br>3min 70% B;<br>6 min 100% B;
B	Agilent HPLC 1260	Zorbax SB-C18 column (4.6 mm×250 mm, 5 μm, Agilent, USA)	0 min 40% B;<br>4min 70% B;<br>13 min 80% B;<br>16 min 100% B;
C	Thermo Fisher Q-Exactive quadrupole Orbitrap mass spectrometer	ACQUITY UPLC BEH C18 column (1.7 μm, 1.0 mm×100 mm)	0 min 20% B;<br>0.5 min 20% B;<br>2 min 25% B;<br>4.5 min 25% B;<br>6 min 50% B;<br>7.5 min 50% B;<br>7.6 min 100% B;<br>9.6 min 100% B

Note: Solvent A, Water containing 0.1% formic acid; Solvent B, Methanol (Method A and Method C) or Acetonitrile (Method B).

The MS parameters were as follows: Sheath gas pressure 45 arb, aux gas pressure 10 arb, discharge voltage 4.5 kV, capillary temperature 350 °C. MS^1 resolution was set as 70,000 FWHM, AGC target $1*E^6$, maximum injection time 50 ms, and scan range *m*/*z* 100–1,000. MS^2 resolution was set as 17,500 FWHM, AGC target $1*E^5$, maximum injection time 100 ms, NCE 35.

Table S2. Primers for candidate genes of PTR.

Primer	Sequence (5′ to 3′)
GuPTR1-F	aggagaaaaaaccccggatccATGACTCAGACACTTATGCATTCACG
GuPTR1-R	agtgagtcgtattacggatccTCAGCGCATGAAATGAATTAGC
GuPTR2-F	aggagaaaaaaccccggatccATGTCTCTAGTCGAATTTGCCCTT
GuPTR2-R	agtgagtcgtattacggatccTTAGCGAATCTTCGGTATGGCT
GuPTR3-F	aggagaaaaaaccccggatccATGGCGTCCAGCGTATTAGAGG
GuPTR3-R	gtgagtcgtattacggatccCTAAACCTTGTGTATAATCAAACTAGCACC
GuPTR4-F	cagcaaatgggtcgcggatccATGGCATCACATGATCAAGAAGC
GuPTR4-R	tgcggccgcaagcttggatccTCACATCTTGTACAACTCAACAACACC
GuPTR5-F	cagcaaatgggtcgcggatccATGGCGAAGGACACCCAAG
GuPTR5-R	tgcggccgcaagcttggatccCTAATTTGATCTTGTAATATGATCGATAAAC
GuPTR6-F	cagcaaatgggtcgcggatccATGGCGGCTTCTTACAACGA
GuPTR6-R	tgcggccgcaagcttggatccTTAAATCCTACTAATTTGAGCAATGAAC

Note: Flanking recombination sequences are shown in lowercase.

Table S3. Primers for GuPTR1 mutants.

Primer	Sequence (5′ to 3′)
G116A-F	gcaATCGATGTTGATCGAACTCATGGAG
G116A-R	AACATCGATtgcAAATTCTGAAGGAAA
I117A-F	gcaGATGTTGATCGAACTCATGGAGT
I117A-R	CAACATCtgcTCCAAATTCTGAAGGAA
K135A-F	gcaGCCAAATTCCGCAGAACAATTGAGG
K135A-R	ATTTGGCtgcAATGTCAAACAAAGCCTT
I117P-F	ccgGATGTTGATCGAACTCATGGAGT
I117P-R	TCAACATCcggTCCAAATTCTGAAGGA
L131A-F	gcaTTTGACATTAAGGCCAAATTCCGCA
L131A-R	ATGTCAAAtgcAGCCTTAGCCCCATCTAC
F132A-F	gcaGACATTAAGGCCAAATTCCGCAGAAC
F132A-R	CCTTAATGTCtgcCAAAGCCTTAGCCCC
K135C-F	tgtGCCAAATTCCGCAGAACAAT
K135C-R	TTTGGCacaAATGTCAAACAAAGCC
K135D-F	gatGCCAAATTCCGCAGAACAATTGAGG
K135D-R	AATTTGGCatcAATGTCAAACAAAGCCTT
K135E-F	gaaGCCAAATTCCGCAGAACAATT
K135E-R	TTTGGCttcAATGTCAAACAAA
K135F-F	tttGCCAAATTCCGCAGAACAATT
K135F-R	ATTTGGCaaaAATGTCAAACAA
K135G-F	ggtGCCAAATTCCGCAGAACAA
K135G-R	TTTGGCaccAATGTCAAACAAA
K135H-F	catGCCAAATTCCGCAGAACAATT
K135H-R	ATTTGGCatgAATGTCAAACAAA
K135I-F	attGCCAAATTCCGCAGAACAATT
K135I-R	TTTGGCaatAATGTCAAACAAAG
K135L-F	ctgGCCAAATTCCGCAGAACAATT
K135L-R	ATTTGGCcagAATGTCAAACAAA
K135M-F	atgGCCAAATTCCGCAGAACAAT
K135M-R	TTTGGCcatAATGTCAAACAAAGC
K135N-F	aatGCCAAATTCCGCAGAACAAT
K135N-R	TTTGGCattAATGTCAAACAAAG
K135P-F	ccgGCCAAATTCCGCAGAACAAT
K135P-R	ATTTGGCcggAATGTCAAACAAAG
K135Q-F	cagGCCAAATTCCGCAGAACAAT
K135Q-R	TGGCctgAATGTCAAACAAAGCCT
K135R-F	cgtGCCAAATTCCGCAGAACAA
K135R-R	TTGGCacgAATGTCAAACAAAGC
K135S-F	agcGCCAAATTCCGCAGAACAA
K135S-R	TTGGCgctAATGTCAAACAAAGC
K135T-F	accGCCAAATTCCGCAGAACAAT
K135T-R	ATTTGGCggtAATGTCAAACAAA
K135V-F	gttGCCAAATTCCGCAGAACAAT
K135V-R	TTTGGCaacAATGTCAAACAAA
K135W-F	tggGCCAAATTCCGCAGAACAA
K135W-R	TTTGGCccaAATGTCAAACAAAGC
K135Y-F	tatGCCAAATTCCGCAGAACAA
K135Y-R	TTTGGCataAATGTCAAACAAAGC

Note: Mutation sites are shown in lowercase.

Table S4. PTR genes for BLAST analysis.

Template genes	Accession numbers in GenBank database
LjPTR1	AB265589.1
LjPTR2	AB265590.1
LjPTR3	AB265591.1
LjPTR4	AB265592.1

Table S5. GenBase accession numbers of GuPTR1–6.

Genes	Accession number
GuPTR1	C_AA110144.1
GuPTR2	C_AA110145.1
GuPTR3	C_AA110146.1
GuPTR4	C_AA110147.1
GuPTR5	C_AA110148.1
GuPTR6	C_AA110149.1

Table S6. Assignment of NMR signals of **3a**.

position	δ_C, type	δ_H (J in Hz)
2	69.5, CH_2	3.92 (1H, m), 4.20 (1H, m)
3	31.0, CH	3.30 (1H, m)
4	30.4, CH_2	2.70(1H, m), 2.89 (1H, m)
5	126.7, CH	6.68 (1H, d, J=8.2 Hz)
6	107.4, CH	6.34 (1H, d, J=8.2 Hz)
7	153.7, C	
8	114.5, C	
9	152.3, C	
10	112.7, C	
1′	119.8, C	
2′	155.9, C	
3′	101.3, CH	6.43 (1H, d, J=2.6 Hz)
4′	158.8, C	
5′	104.3, CH	6.34 (1H, d, J=8.5, 2.6 Hz)
6′	127.7, CH	6.99 (1H, d, J=8.5 Hz)
1″	22.0, CH_2	3.18 (2H, d, J=7.1 Hz)
2″	123.5, CH	5.14 (1H, t, J=7.1 Hz)
3″	129.6, C	
4″	25.5, CH_3	1.61 (3H, s)
5″	17.7, CH_3	1.69 (3H, s)
4′-OCH_3	54.8, CH_3	3.66 (3H, s)

Table S7. Catalytic activities of GuPTR1–6 utilizing **1**–**16**.

Substrate	Relative Conversion Rate (%)					
	GuPTR1	GuPTR 2	GuPTR 3	GuPTR 4	GuPTR 5	GuPTR 6
**1**	95.35 ±0.58	17.41 ±0.43	18.79 ±1.34	93.73 ±3.08	18.72 ±1.35	90.22 ±2.69
**2**	98.28 ±0.27	1.22 ±0.23	2.02 ±0.17	85.54 ±0.10	1.85 ±0.32	39.94 ±0.30
**3**	87.34 ±0.10	4.30 ±0.52	0.00	8.21 ±1.14	0.00	87.11 ±0.09
**4**	90.70 ±1.58	29.06 ±1.36	0.00	56.87 ±3.61	0.00	83.87 ±3.97
**5**	56.26 ±1.32	0.00	0.00	0.00	0.00	59.30 ±3.49
**6**	86.55 ±1.58	0.00	0.00	0.00	0.00	74.38 ±2.48
**7**	94.31 ±0.05	0.00	0.00	0.00	0.00	50.20 ±3.02
**8**	78.74 ±1.79	0.00	0.00	0.00	0.00	40.90 ±0.33
**9**	97.17 ±2.69	0.00	0.00	0.00	0.00	6.51 ±0.64
**10**	98.79 ±0.66	0.00	0.00	0.00	0.00	97.83 ±0.29
**11**	0.00	0.00	0.00	0.00	0.00	0.00
**12**	0.00	0.00	0.00	0.00	0.00	0.00
**13**	0.00	0.00	0.00	0.00	0.00	0.00
**14**	0.00	0.00	0.00	0.00	0.00	0.00
**15**	0.00	0.00	0.00	0.00	0.00	0.00
**16**	0.00	0.00	0.00	0.00	0.00	0.00

Note: Data are presented as mean ± SD, $n = 3$ (three independent samples were tested).

Table S8. Data collection, and refinement statistics of crystal structure.

PDB ID	9UM2
Space group	$C222_1$
Cell parameters	
a, b, c (Å)	82.56, 108.79, 77.31
α, β, γ (°)	90, 90, 90
Resolution (Å)	38.65 – 2.10 (2.10–2.16)[a]
R_{merge} (%)	5.9 (14.3)
$CC_{1/2}$ (%)	98.3 (89.6)
$I/\sigma I$	13.5 (5.1)
Completeness (%)	98.2 (99.6)
Average redundancy	1.9 (1.9)
**Refinement**	
No. of measured reflections	38801 (3186)
No. of unique reflections	20558 (1652)
R_{work}/R_{free}(%)	18.17/21.88
B factors (Å^2)	27.40
Rmsd bond lengths (Å)	0.01
Rmsd bond angles (°)	1.35
Ramachandran plot residues (%)	
Favored	95.89
Allowed	4.11
Outliers	0.00

[a] Values in parentheses are for highest-resolution shell.

Table S9. Catalytic activities of GuPTR1-mutants utilizing **1**.

Mutants	Conversion rate (%)	Standard deviation
Wild type	95.22	3.80
G116A	8.56	0.23
I117A	98.39	1.09
I117P	2.00	0.38
L131A	75.43	0.92
K135A	0.85	0.00
K135C	1.63	0.21
K135D	0.89	0.03
K135E	6.40	0.46
K135F	1.23	0.12
K135G	1.35	0.32
K135H	2.33	0.04
K135I	1.16	0.15
K135L	1.28	0.11
K135M	5.27	0.23
K135N	1.13	0.10
K135P	1.19	0.00
K135Q	1.03	0.05
K135R	3.38	0.15
K135S	1.93	0.38
K135T	2.31	0.23
K135V	1.25	0.30
K135W	2.22	1.70
K135Y	1.35	0.08
F132A	2.15	0.06

Note: $n = 3$, three independent samples were tested.

Table S10. Catalytic activities of GuPTR2–6 and related K-A mutants utilizing **1**.

Mutants	Relative conversion rate (%)	Standard deviation
GuPTR2-WT	17.41	0.43
GuPTR2-K137A	1.40	0.00
GuPTR3-WT	18.79	1.34
GuPTR3-K135A	1.36	0.08
GuPTR4-WT	93.73	3.08
GuPTR4-K133A	1.34	0.01
GuPTR5-WT	18.72	1.35
GuPTR5-K134A	1.21	0.07
GuPTR6-WT	90.22	2.69
GuPTR6-K133A	1.53	0.07

Note: $n = 3$, three independent samples were tested.

Table S11. Catalytic activities of MnPTR, TcPTR, TpPTR1, TpPTR2 and their mutants utilizing **1**.

Mutants	Relative conversion rate (%)	Standard deviation
MnPTR-WT	78.44	2.96
MnPTR-K133A	12.83	2.15
TcPTR-WT	97.70	0.28
TcPTR-K133A	2.25	0.56
TpPTR1-WT	69.02	0.94
TpPTR1-K133C	1.76	0.32
TpPTR2-WT	4.33	1.11
TpPTR2-C133K	100.00	0.00

Note: $n = 3$, three independent samples were tested.

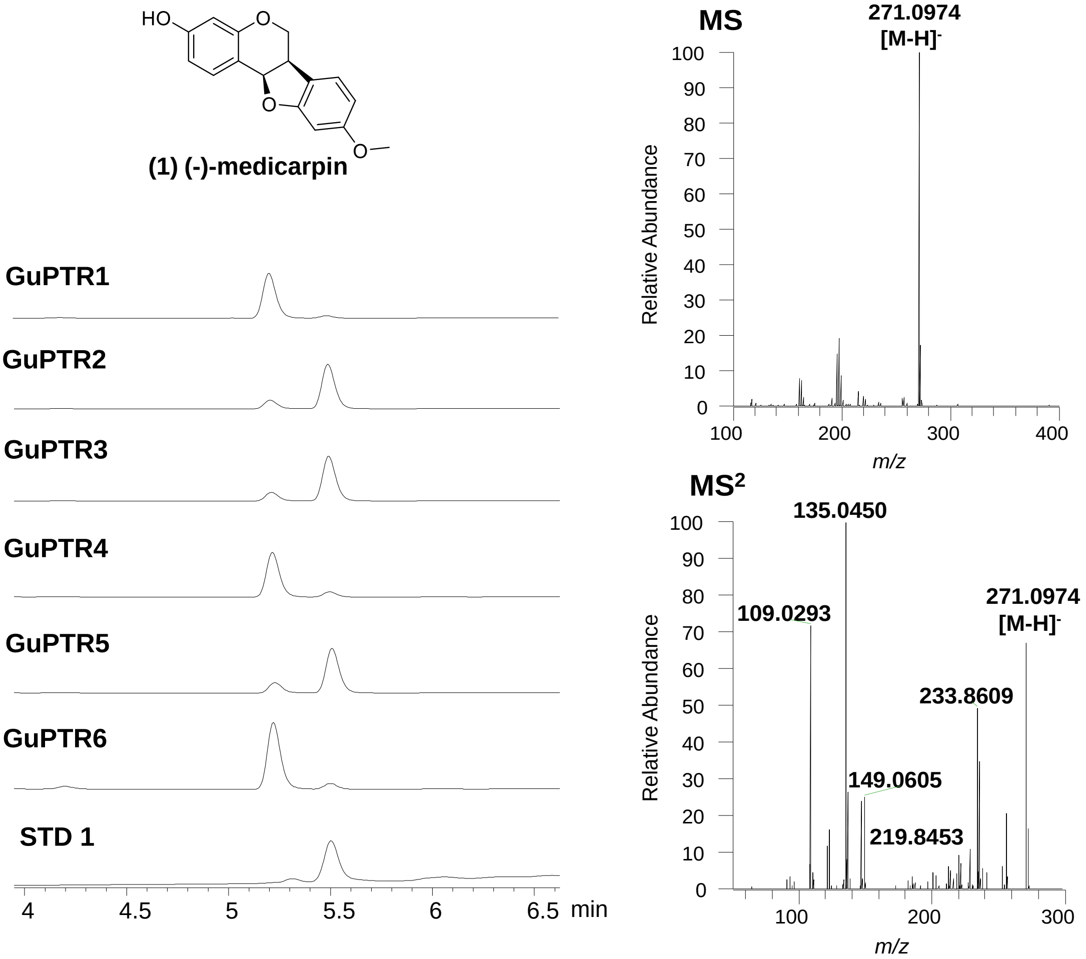

Figure S1. Catalytic activities of GuPTR1–6 toward **1**. Shown are the structure of the substrate, HPLC/UV chromatograms of the reaction mixtures (λ=280 nm), and (–)-ESI-MS and MS/MS spectra of the product.

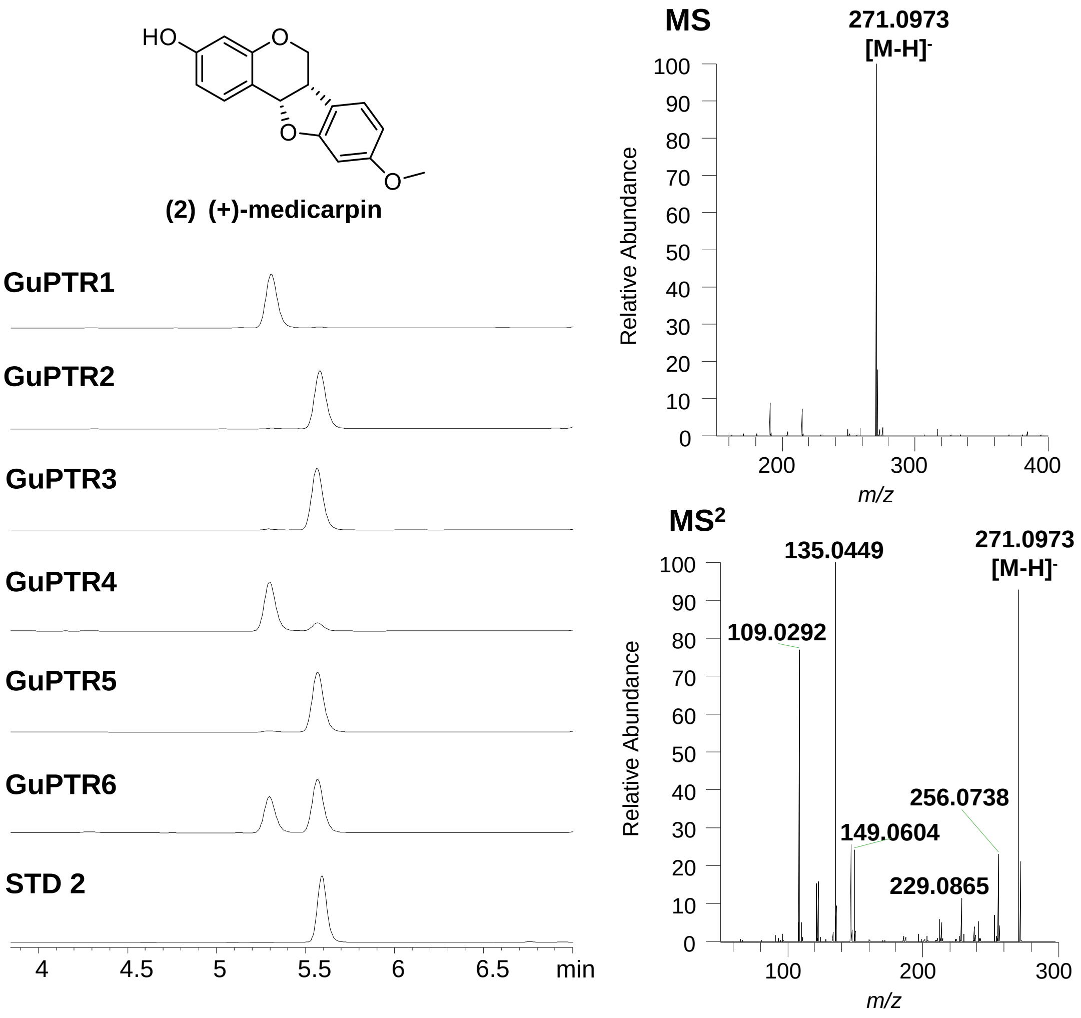

Figure S2. Catalytic activities of GuPTR1–6 toward **2**. Shown are the structure of the substrate, HPLC/UV chromatograms of the reaction mixtures (λ=280 nm), and (–)-ESI-MS and MS/MS spectra of the product.

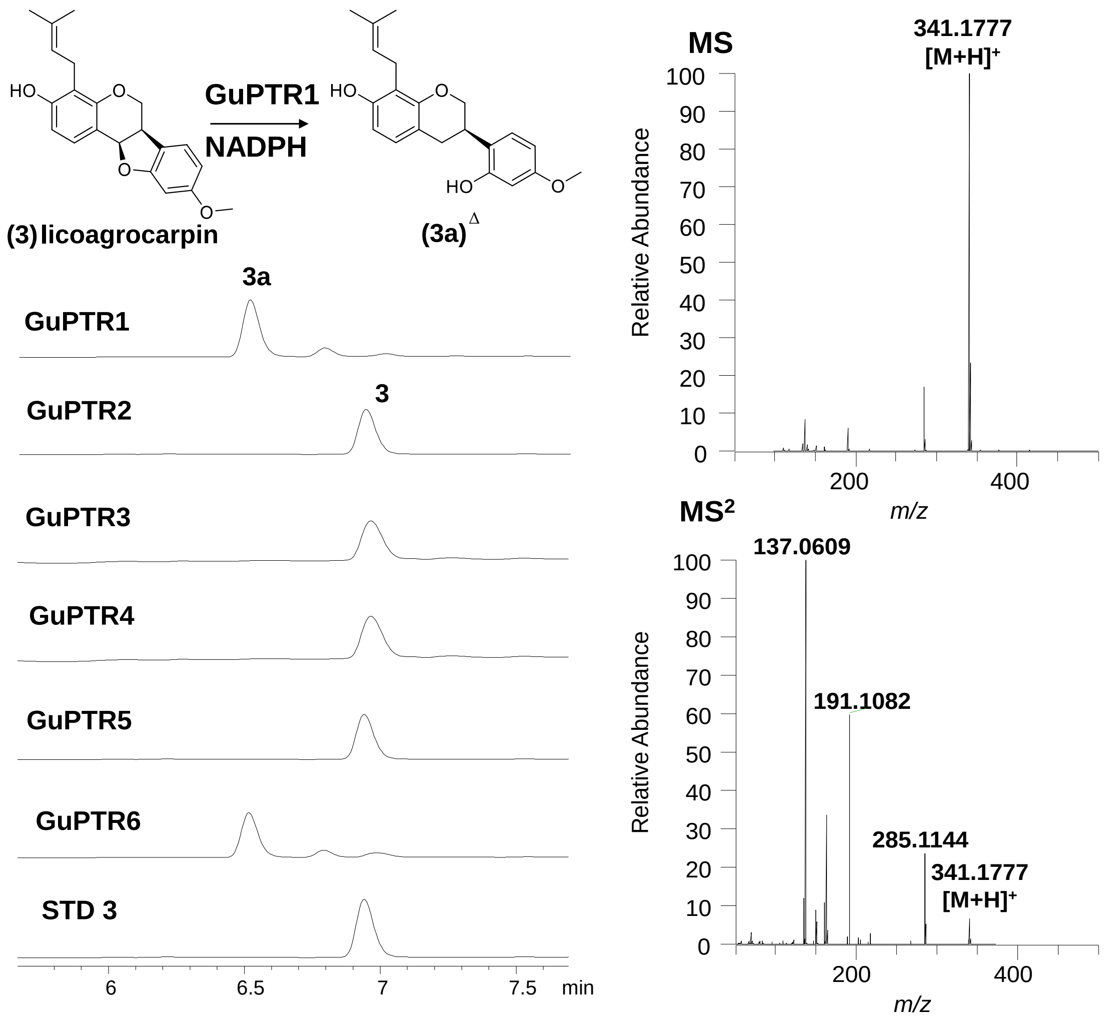

Figure S3. Catalytic activities of GuPTR1–6 toward **3**. Shown are the structure of the substrate and product, HPLC/UV chromatograms of the reaction mixtures (λ=280 nm), and (–)-ESI-MS and MS/MS spectra of the product. $^{\Delta}$, the product was purified in this work and was identified by NMR analysis.

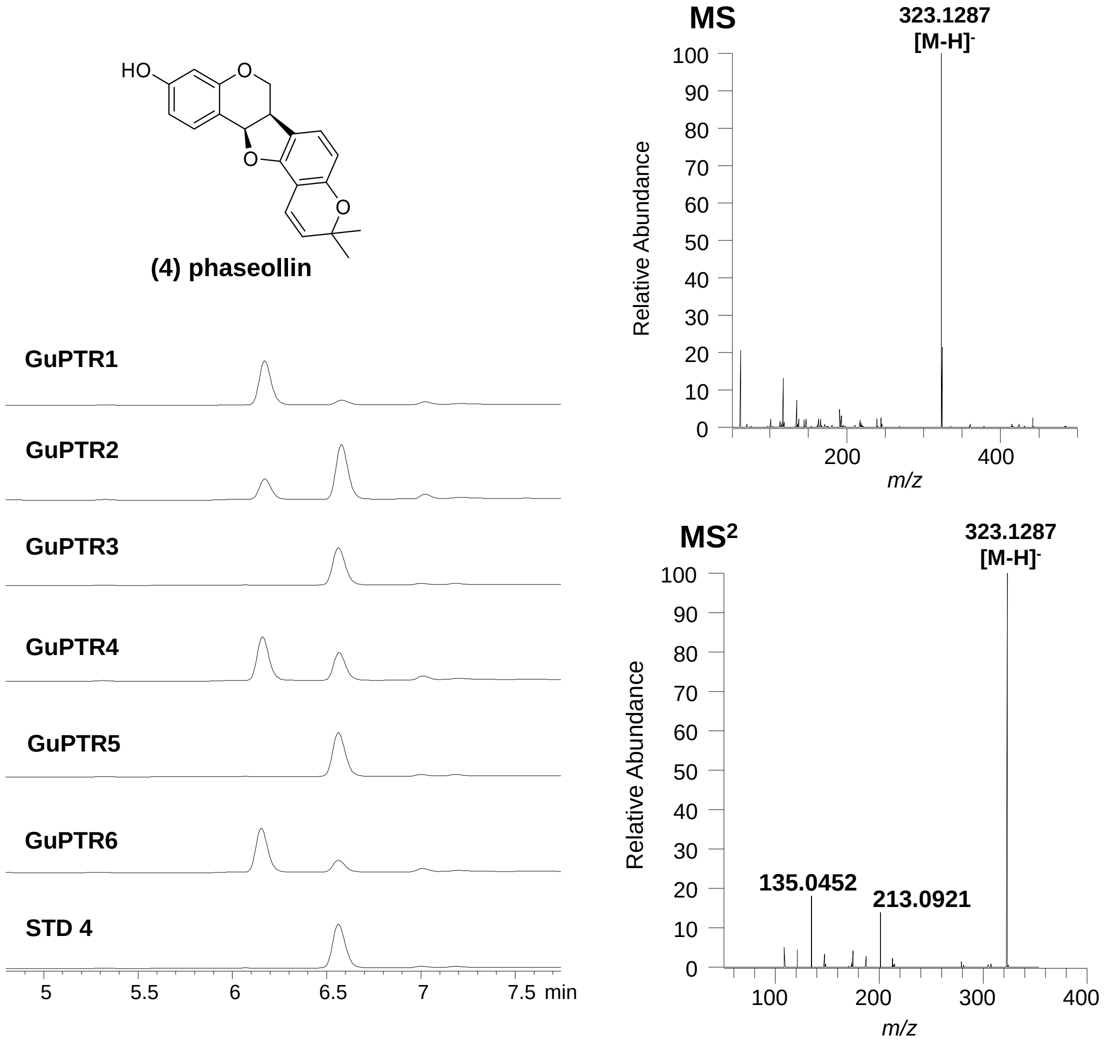

Figure S4. Catalytic activities of GuPTR1–6 toward **4**. Shown are the structure of the substrate, HPLC/UV chromatograms of the reaction mixtures (λ=280 nm), and (–)-ESI-MS and MS/MS spectra of the product.

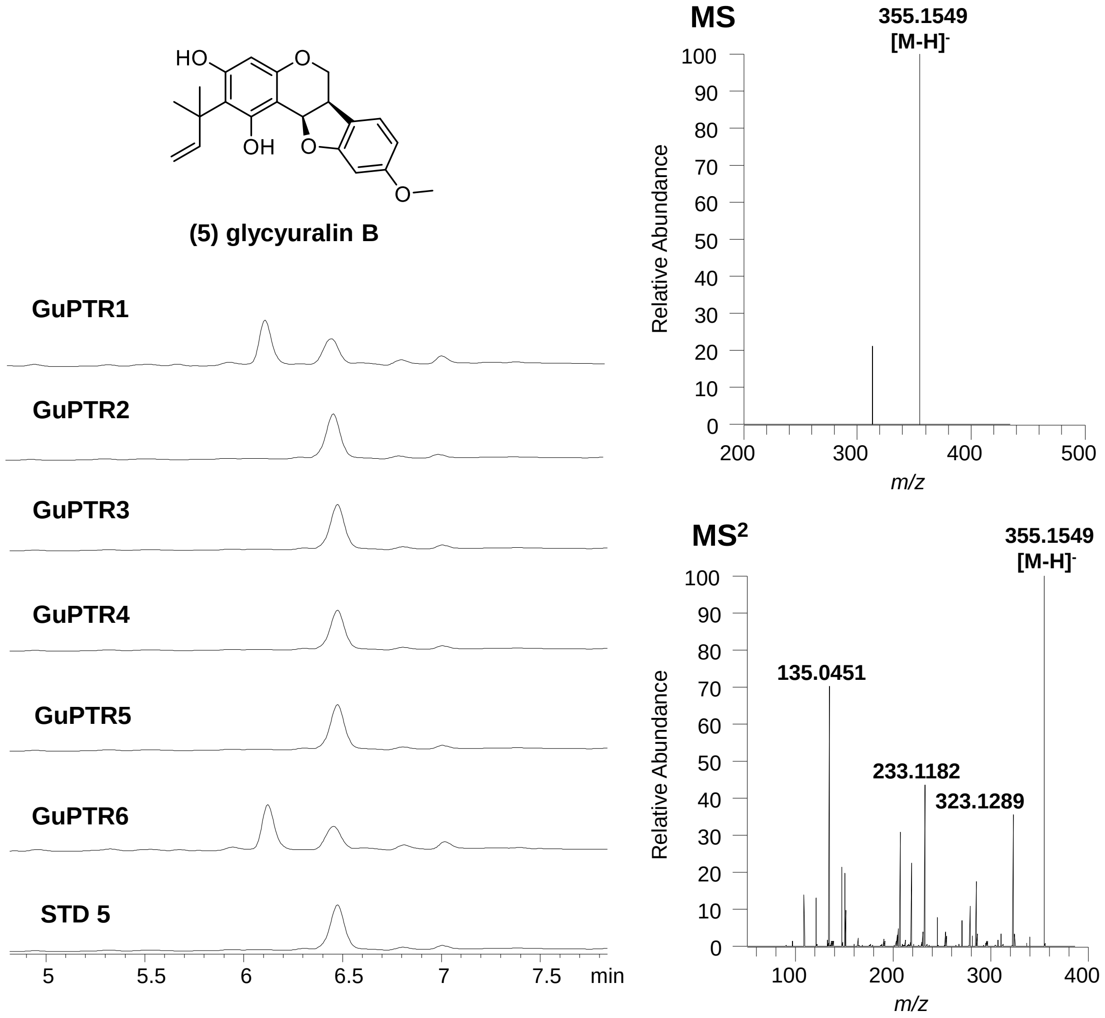

Figure S5. Catalytic activities of GuPTR1–6 toward **5**. Shown are the structure of the substrate, HPLC/UV chromatograms of the reaction mixtures (λ=280 nm), and (–)-ESI-MS and MS/MS spectra of the product.

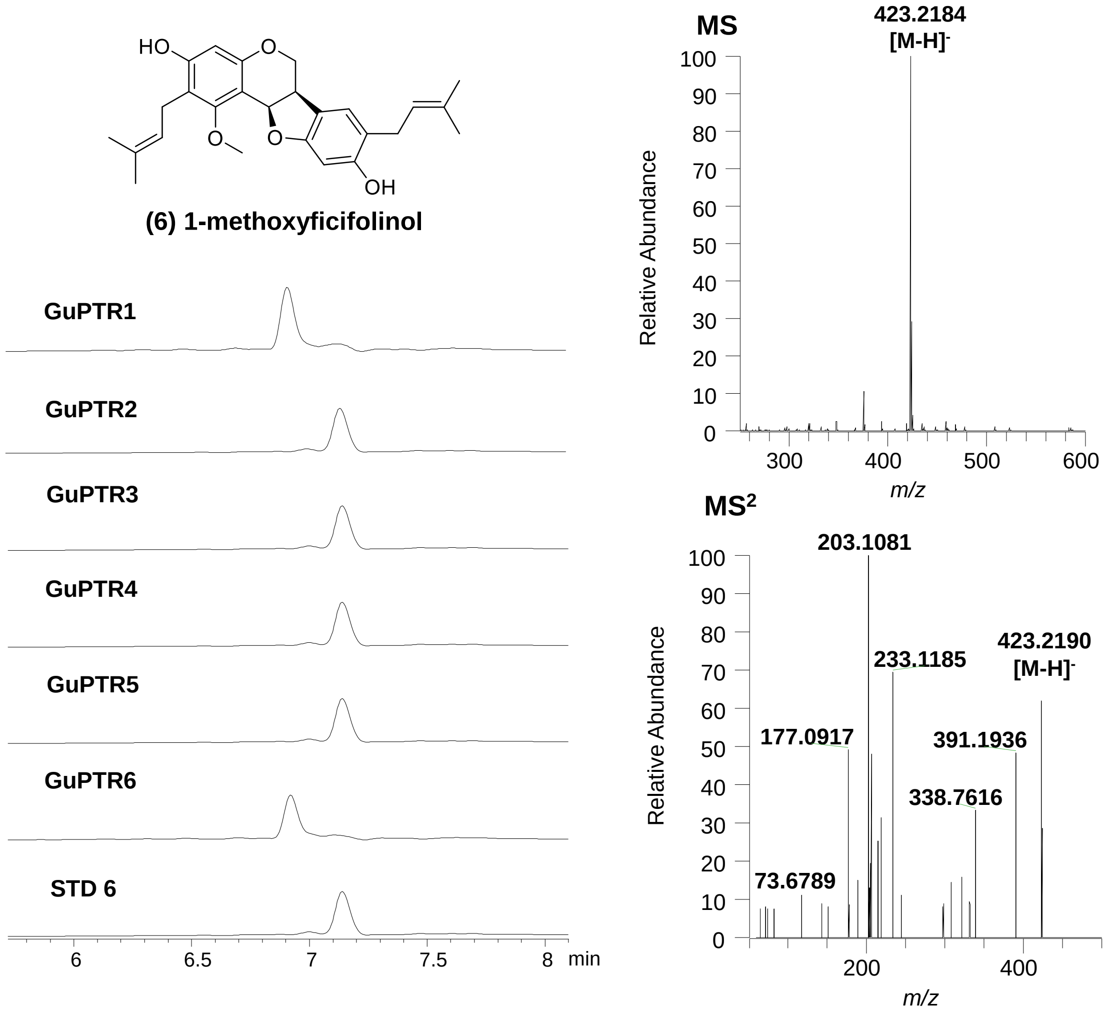

Figure S6. Catalytic activities of GuPTR1–6 toward **6**. Shown are the structure of the substrate, HPLC/UV chromatograms of the reaction mixtures (λ=280 nm), and (–)-ESI-MS and MS/MS spectra of the product.

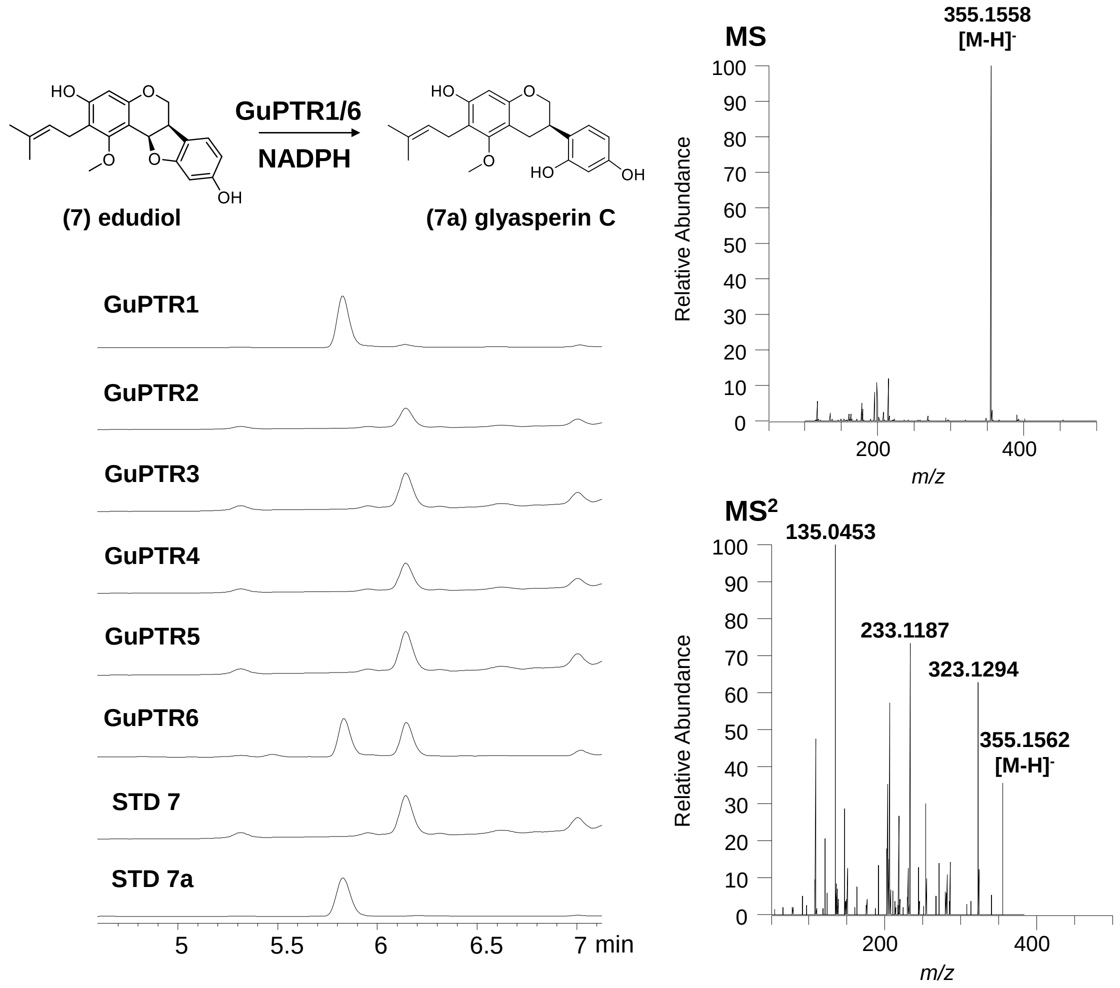

Figure S7. Catalytic activities of GuPTR1–6 toward **7**. Shown are the structure of the substrate and product, HPLC/UV chromatograms of the reaction mixtures (λ=280 nm), and (–)-ESI-MS and MS/MS spectra of the product. The product was identified as glyasperin C (**7a**) by comparing with a reference standard.

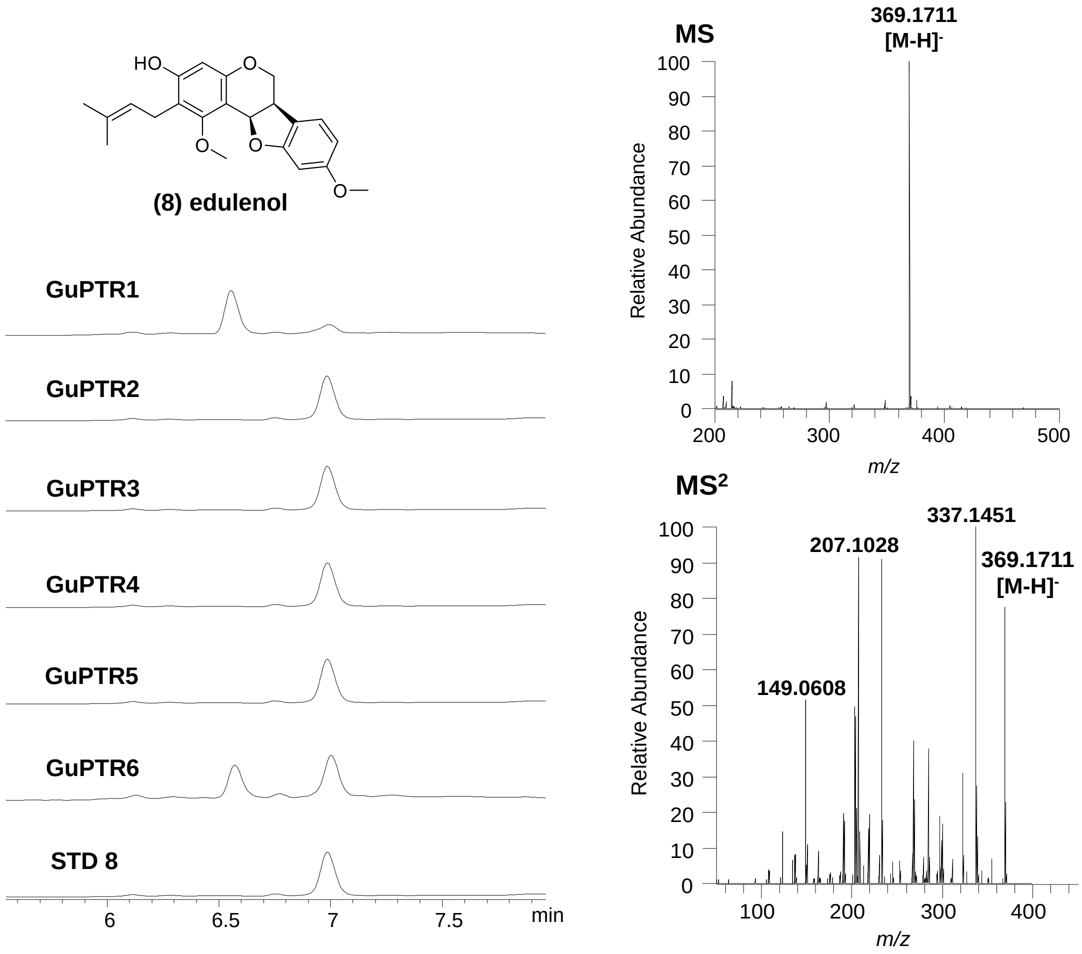

Figure S8. Catalytic activities of GuPTR1–6 toward **8**. Shown are the structure of the substrate, HPLC/UV chromatograms of the reaction mixtures (λ=280 nm), and (–)-ESI-MS and MS/MS spectra of the product.

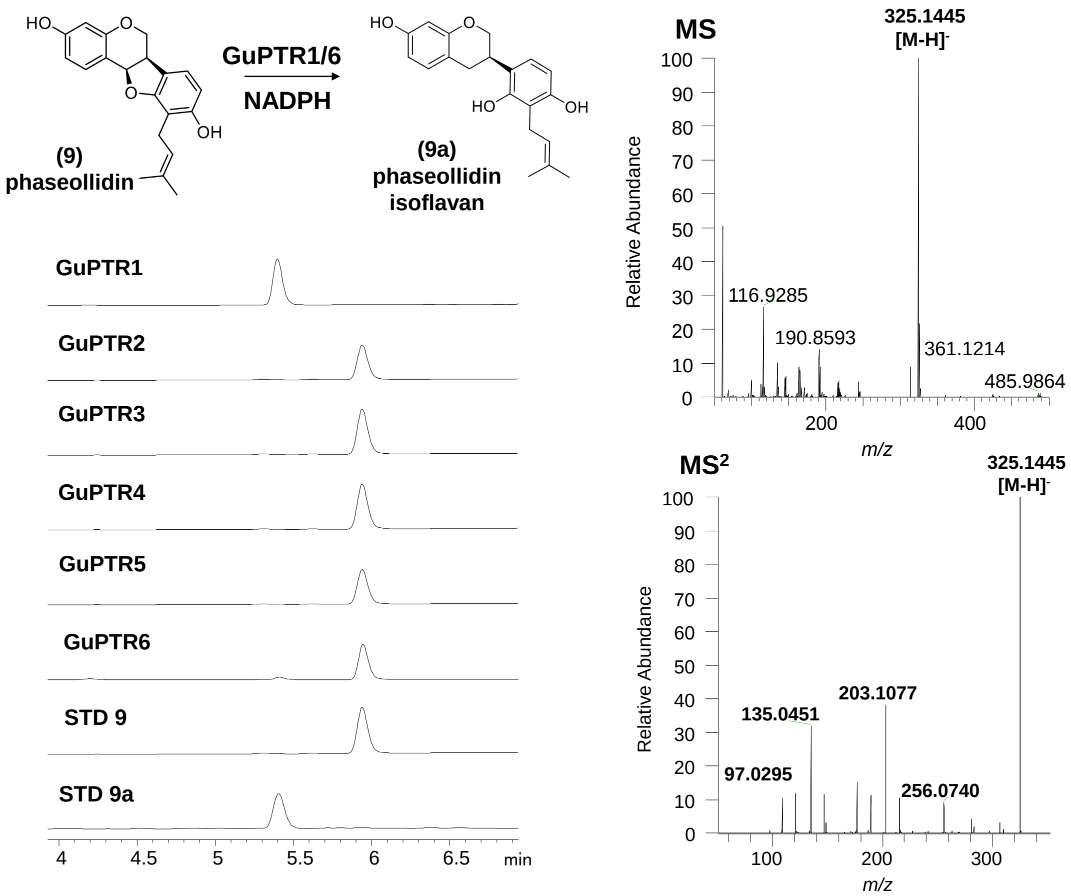

Figure S9. Catalytic activities of GuPTR1–6 toward **9**. Shown are the structure of the substrate and product, HPLC/UV chromatograms of the reaction mixtures (λ=280 nm), and (–)-ESI-MS and MS/MS spectra of the product. The product was identified as phaseollidin isoflavan (**9a**) by comparing with a reference standard.

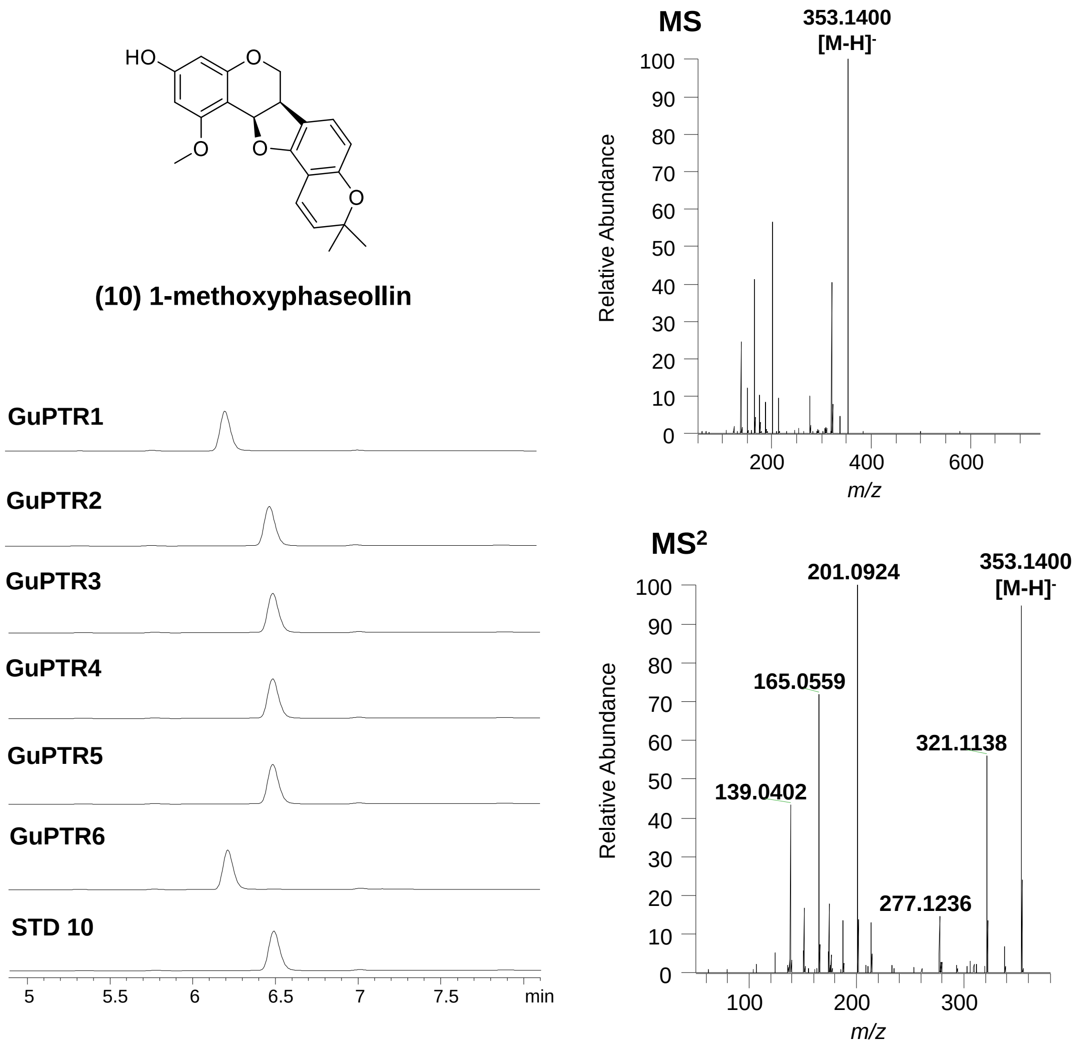

Figure S10. Catalytic activities of GuPTR1–6 toward **10**. Shown are the structure of the substrate, HPLC/UV chromatograms of the reaction mixtures (λ=280 nm), and (–)-ESI-MS and MS/MS spectra of the product.

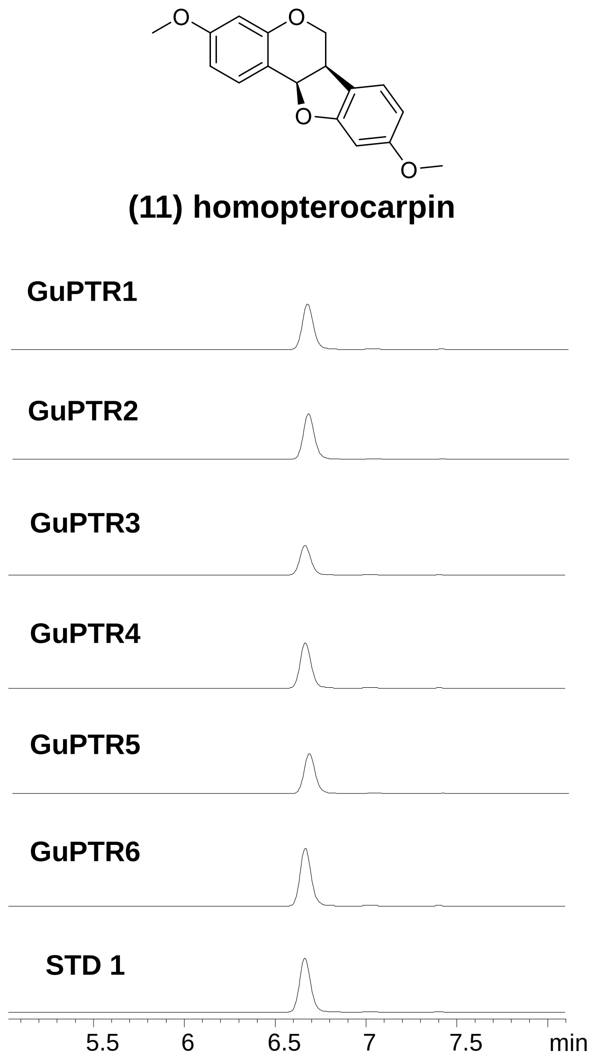

Figure S11. Catalytic activities of GuPTR1–6 toward **11**. Shown are the structure of the substrate and the HPLC/UV chromatograms of the reaction mixtures (λ=280 nm).

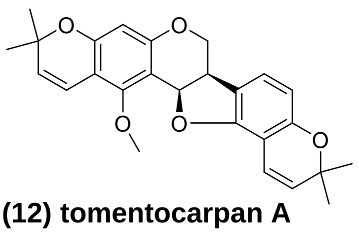

(12) tomentocarpan A

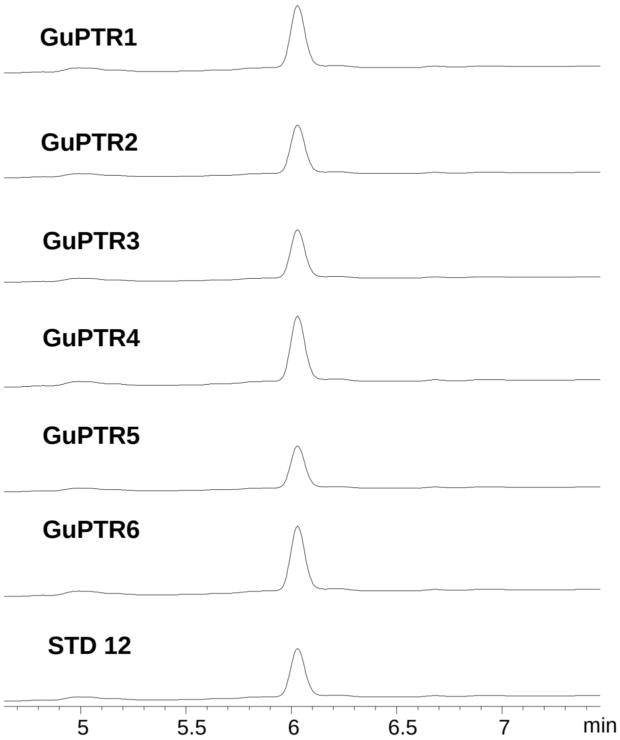

Figure S12. Catalytic activities of GuPTR1–6 toward **12**. Shown are the structure of the substrate and the HPLC/UV chromatograms of the reaction mixtures (λ=280 nm).

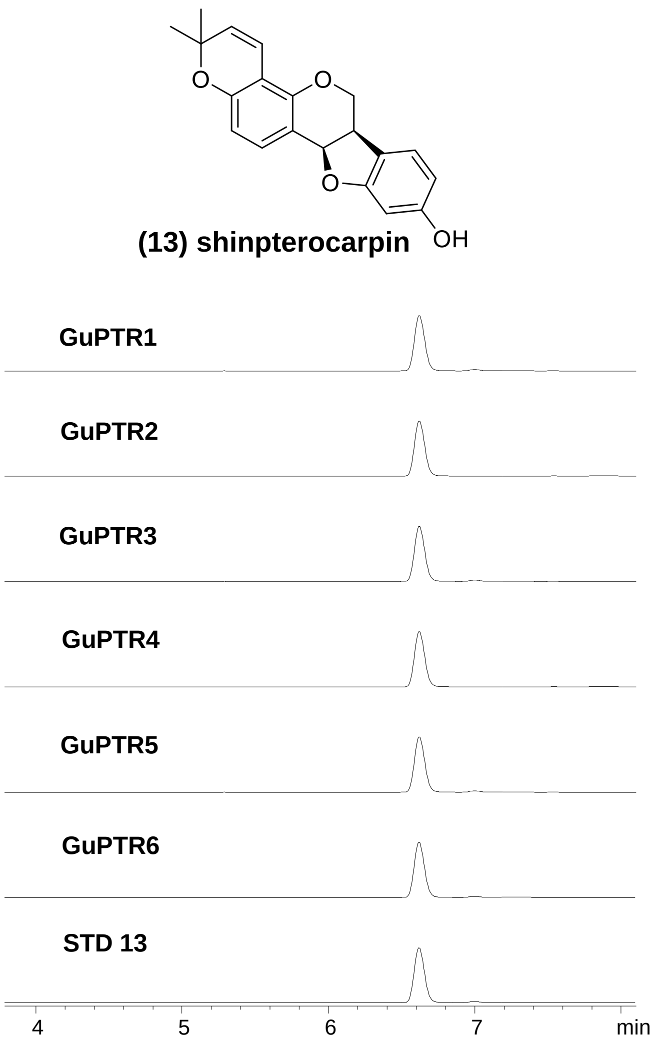

Figure S13. Catalytic activities of GuPTR1–6 toward **13**. Shown are the structure of the substrate and the HPLC/UV chromatograms of the reaction mixtures (λ=280 nm).

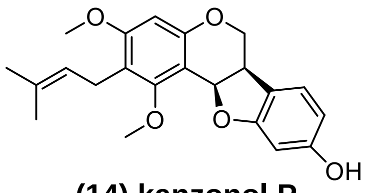

(14) kanzonol P

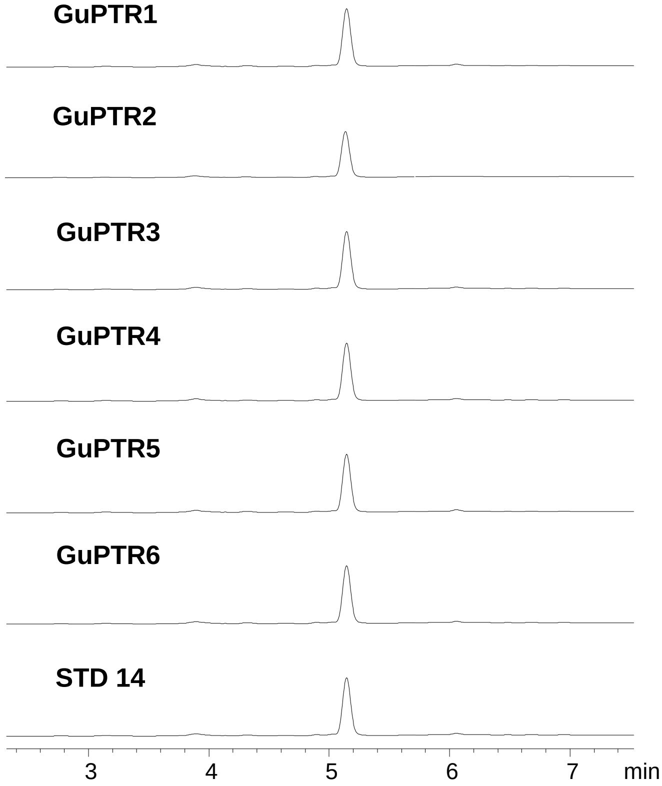

Figure S14. Catalytic activities of GuPTR1–6 toward **14**. Shown are the structure of the substrate and the HPLC/UV chromatograms of the reaction mixtures (λ=280 nm).

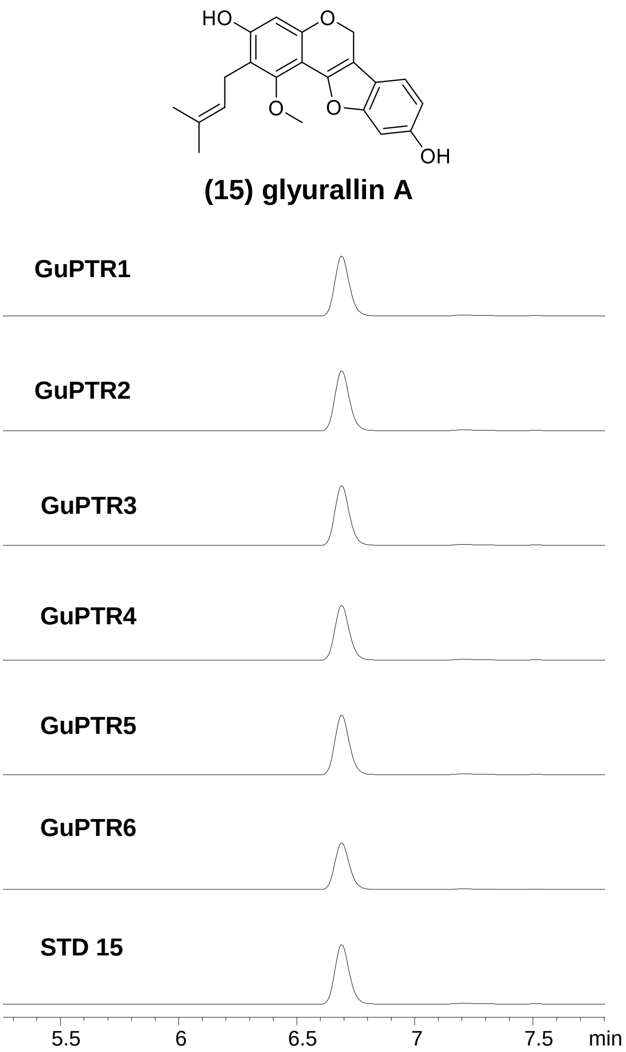

Figure S15. Catalytic activities of GuPTR1–6 toward **15**. Shown are the structure of the substrate and the HPLC/UV chromatograms of the reaction mixtures (λ=365 nm).

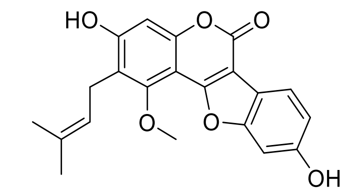

(16) glycyrol

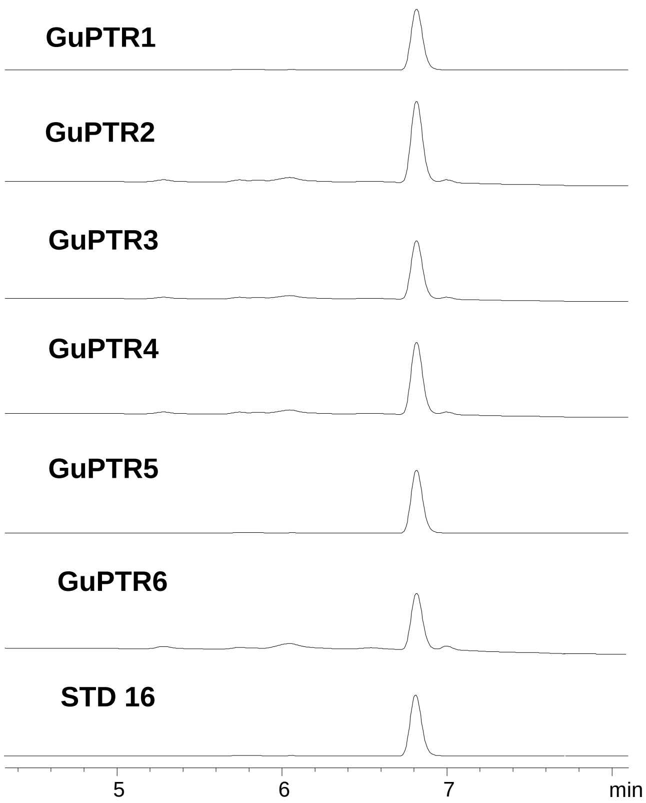

Figure S16. Catalytic activities of GuPTR1–6 toward **16**. Shown are the structure of the substrate and the HPLC/UV chromatograms of the reaction mixtures (λ=254 nm).

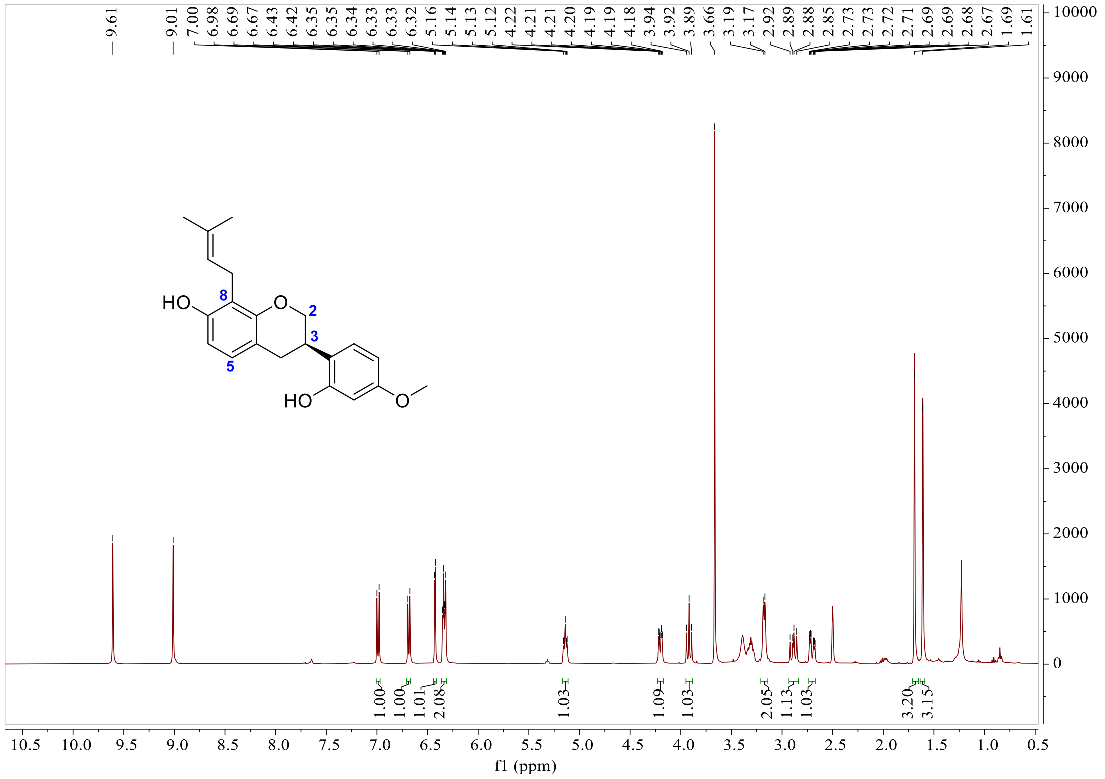

Figure S17. ^{1}H NMR spectrum of **3a** in DMSO-d_6 (400 MHz).

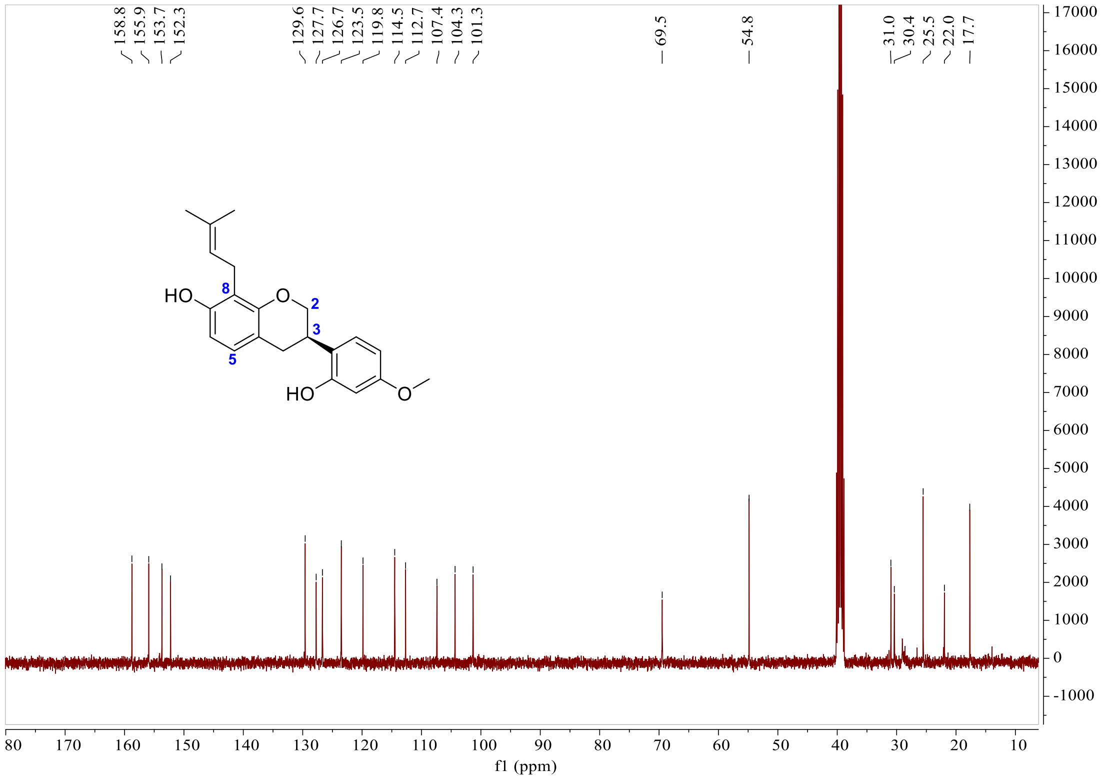

Figure S18. ^{13}C NMR spectrum of **3a** in DMSO-d_6 (100 MHz).

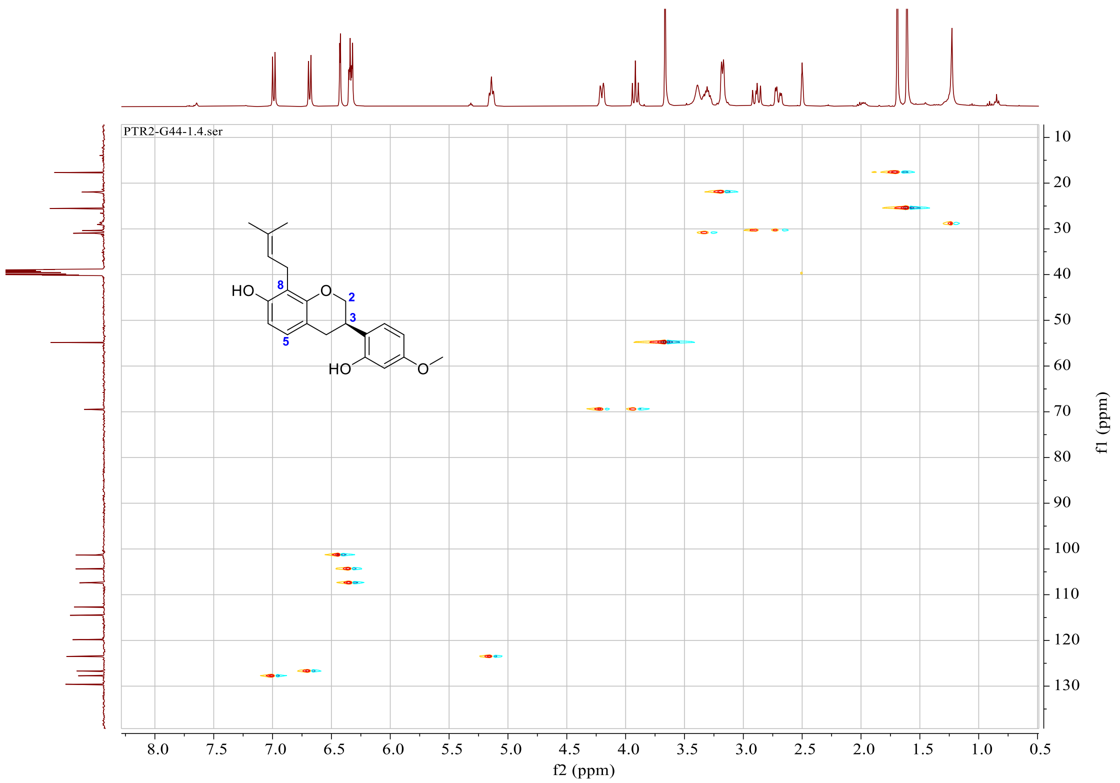

Figure S19. HSQC spectrum of **3a** in DMSO-d_6 (400 MHz).

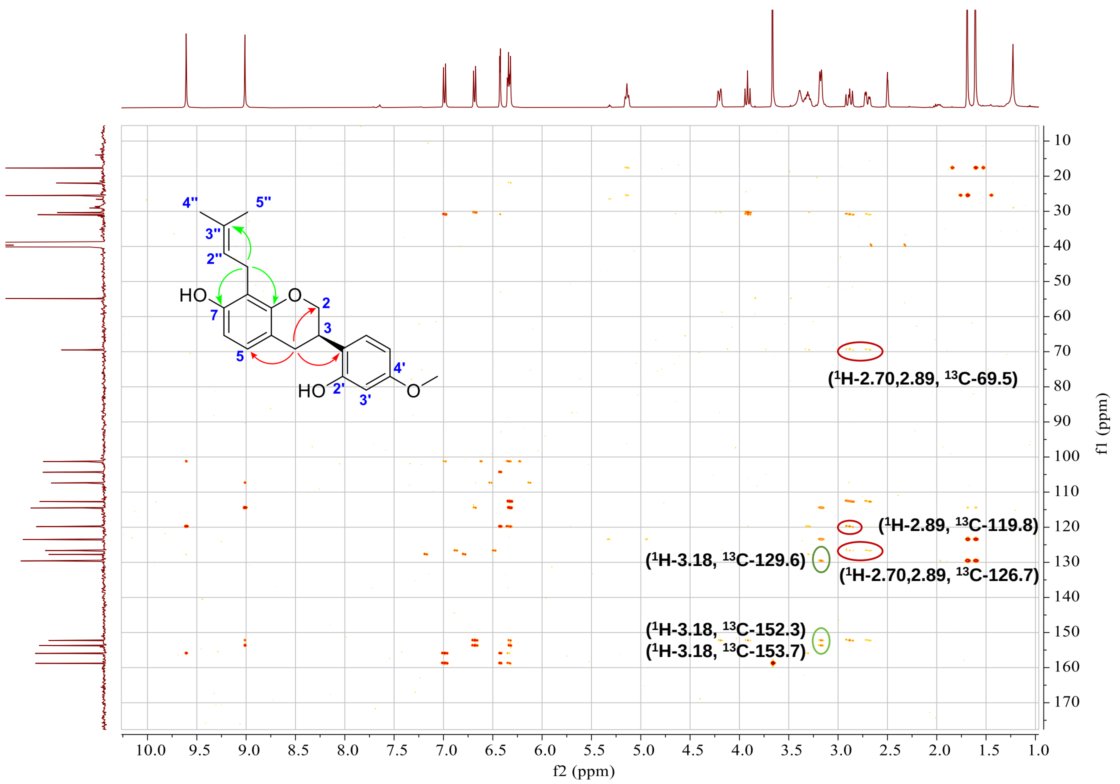

Figure S20. HMBC spectrum of **3a** in DMSO-d_6 (400 MHz).

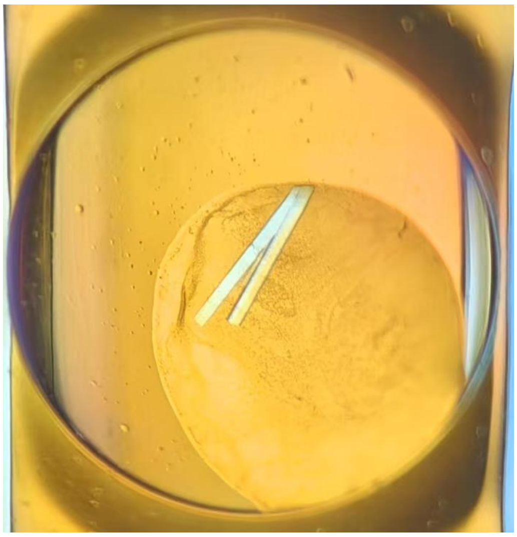

Figure S21. Crystal picture of GuPTR1.

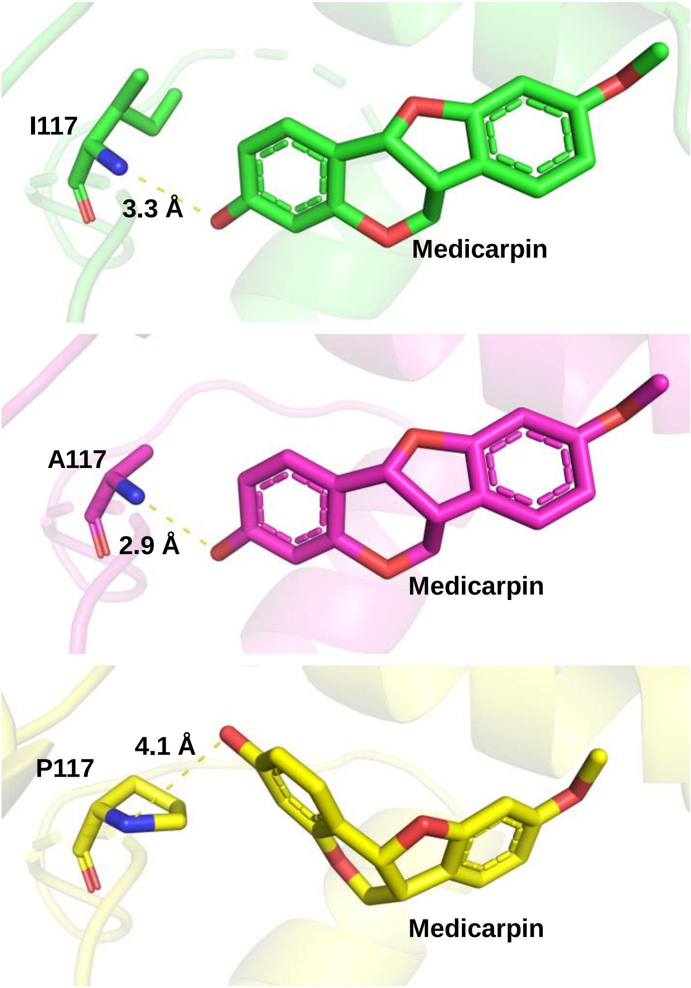

Figure S22. Interactions between the three amino acid residues at position 117 and medicarpin.

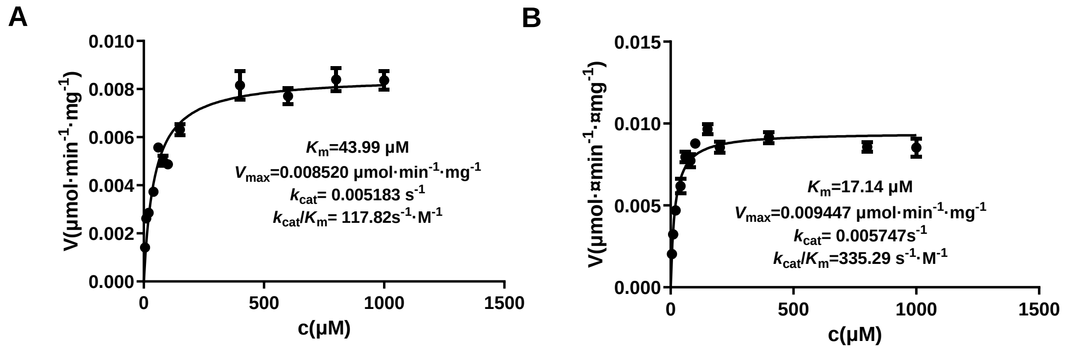

Figure S23. Kinetic analysis for GuPTR1$_{K135A}$ (**A**) and GuPTR1$_{I117P}$ (**B**) against **1**.

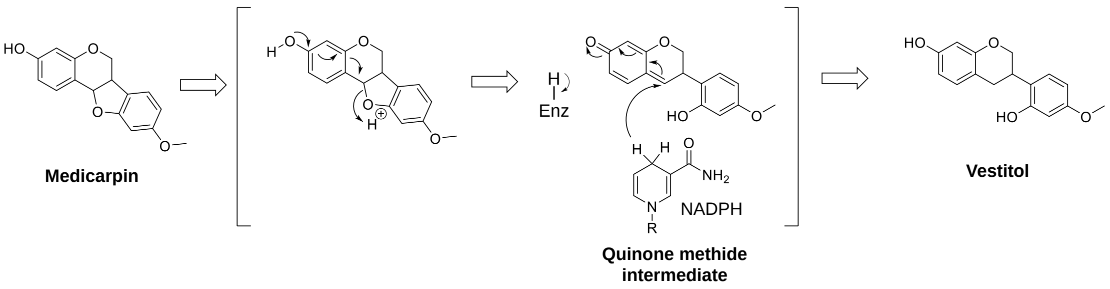

Figure S24. Putative reaction scheme of PTRs in previous work[1].

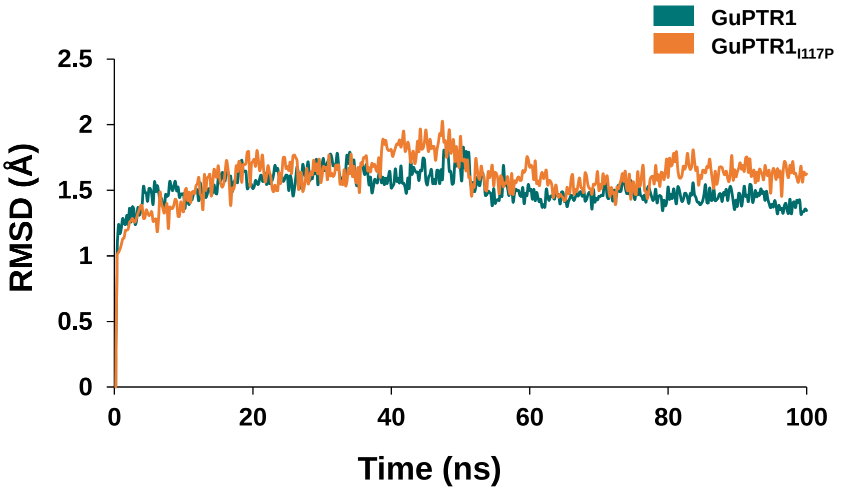

Figure S25. RMSD of protein structures of GuPTR1 and GuPTR1$_{I117P}$ within 100 ns (*n*=3, data were obtained from three independent technical replicates).

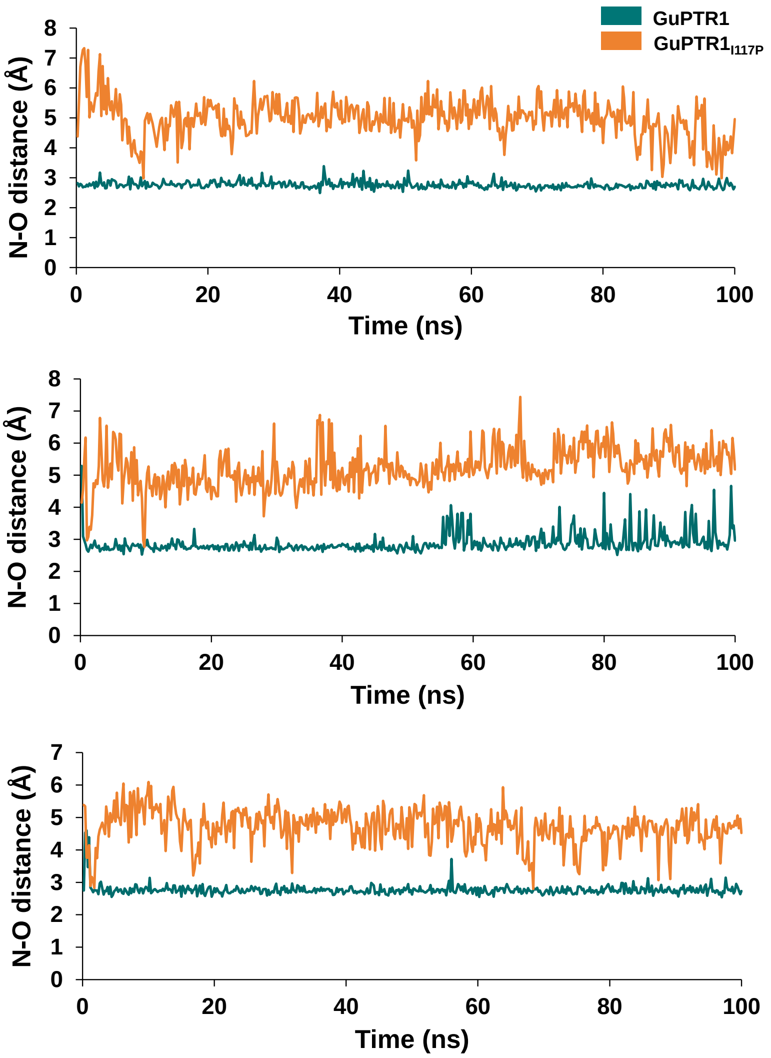

Figure S26. Three independent molecular dynamics of N–O distance of GuPTR1$_{I117P}$ mutant and wild type GuPTR1 in 100 ns.

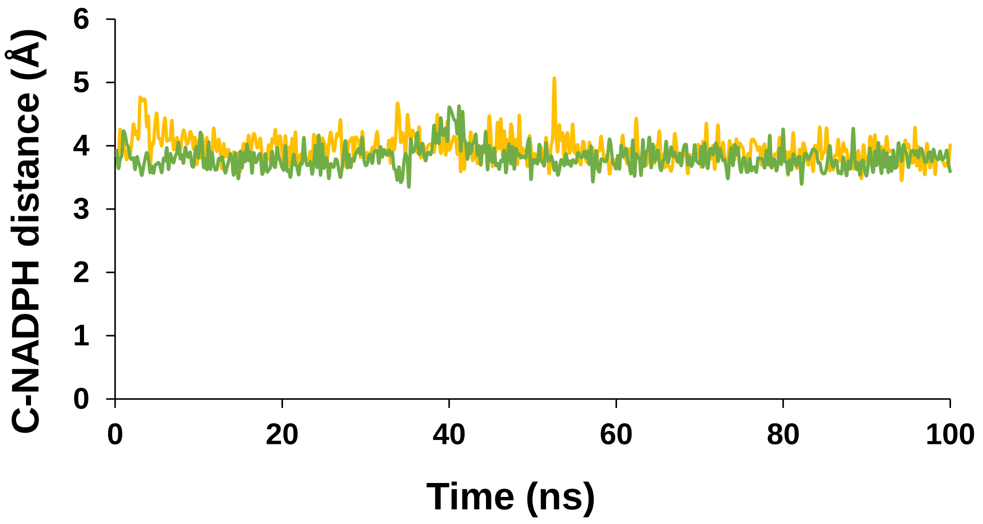

Figure S27. Distance between the C-4 atom on the substrate and the hydride-donating site of NADPH in 100 ns MD simulation of GuPTR1$_{I117P}$ mutant and GuPTR1 (*n*=3, data were obtained from three independent technical replicates).

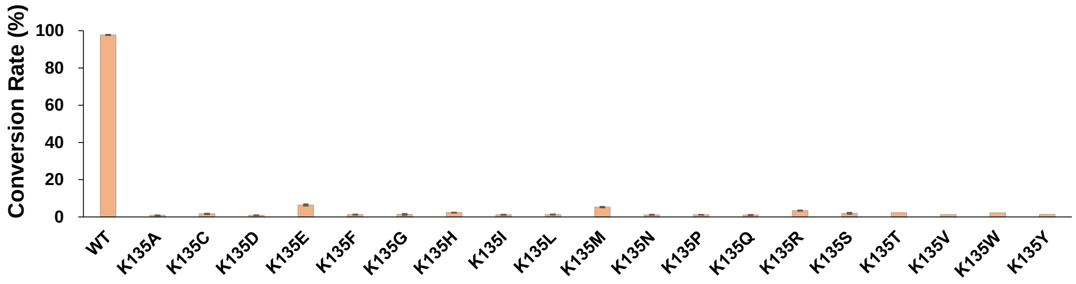

Figure S28. Site-directed saturation mutagenesis screening of K135 (*n*=3). Compound **1** was used as the substrate.

						135							
GuPTR1	A	L	F	D	I	K	A	K	F	R	R	T
GuPTR2	S	A	F	A	T	K	A	K	I	R	R	T
GuPTR3	A	L	F	D	I	K	A	K	F	R	R	T
GuPTR4	D	L	F	D	Y	K	V	K	I	R	R	T
GuPTR5	T	T	Y	Q	V	K	A	K	L	R	R	A
GuPTR6	S	A	F	A	I	K	V	Q	I	R	R	T
LjPTR1-AB265589	A	I	F	E	S	K	S	K	I	R	R	A
LjPTR2-AB265590	E	L	F	D	T	K	V	N	I	R	R	T
LjPTR3-AB265591	S	T	Y	Q	V	K	V	N	V	R	R	A
LjPTR4-AB265592	S	A	F	A	A	K	A	H	I	R	R	T
PCBER-AJ005803	T	A	F	A	M	K	A	Q	I	R	R	A
PCBER-HE574559	S	V	F	E	L	K	A	K	V	R	R	A
PLR-JF264893	E	T	F	D	Q	K	L	E	V	R	N	A
PLR-ABM68630	E	T	F	D	Q	K	M	V	V	R	K	A
PLR-CAH60858	E	T	F	D	L	K	M	V	V	R	K	A
PLR-AAF63507	A	T	F	D	E	K	M	V	V	R	K	A
PLR-AAF63508	I	V	F	I	D	K	I	K	V	R	E	A

Figure S29. Sequence alignment of PTRs with PCBERs and PLRs.

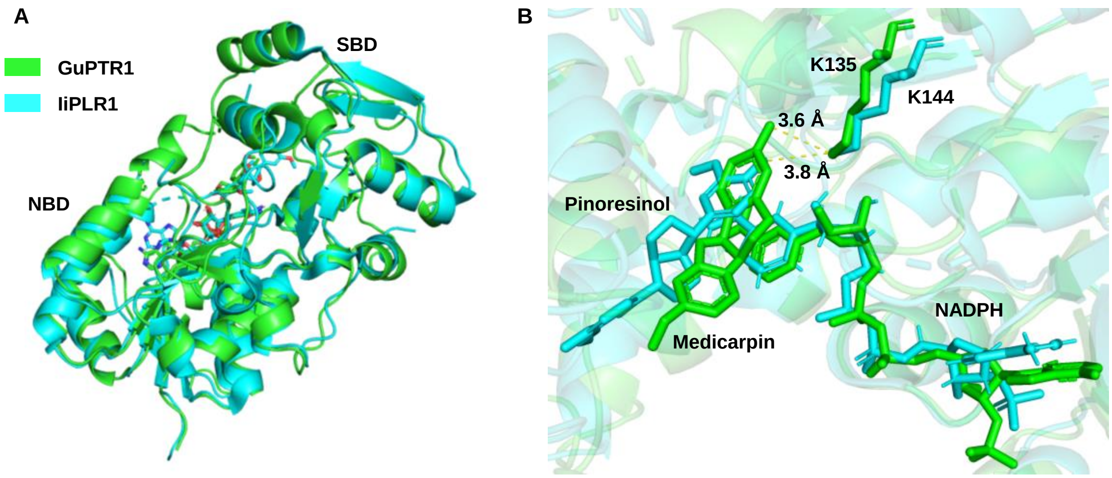

Figure S30. Structural alignment of GuPTR1 and IiPLR1. (**A**) Alignment of crystal structures of GuPTR1 and IiPLR1 (PDB ID: 7CS4). (**B**) Alignment of relative position of K135/K144 and ligands in the structures of GuPTR1 and IiPLR1.

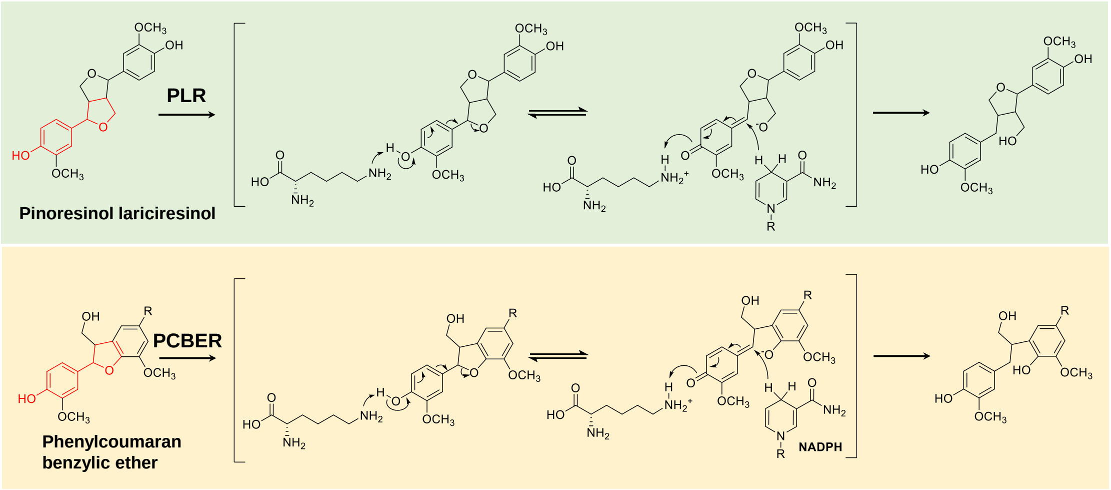

Figure S31. Putative catalytic mechanisms of PLR and PCBER.

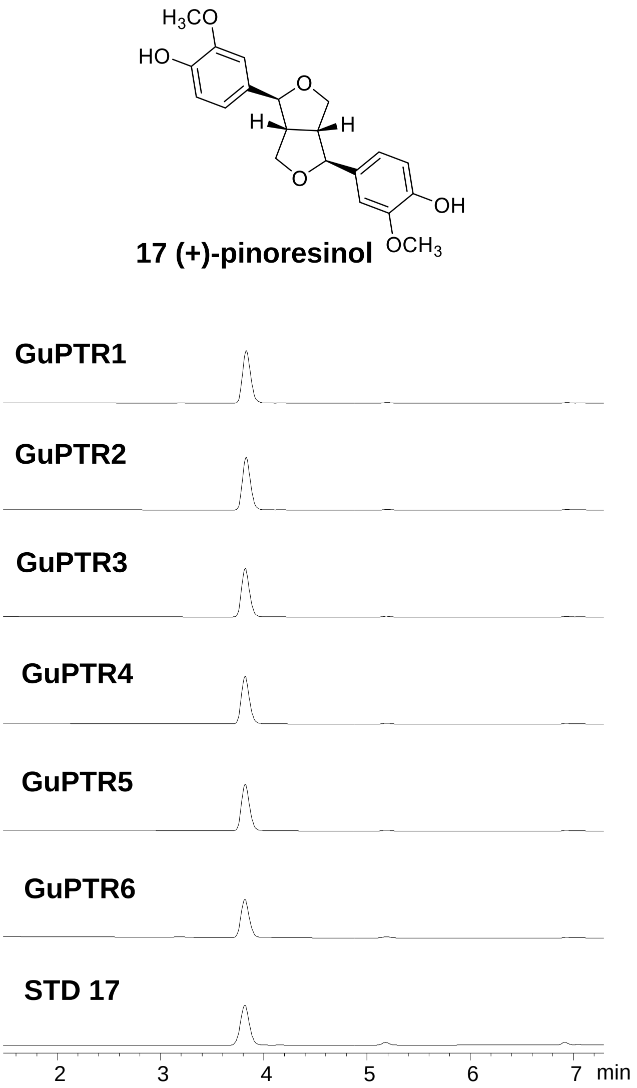

Figure S32. Catalytic activities of GuPTR1–6 toward **17**. Shown are the structure of the substrate and the HPLC/UV chromatograms of the reaction mixtures (λ=280 nm).

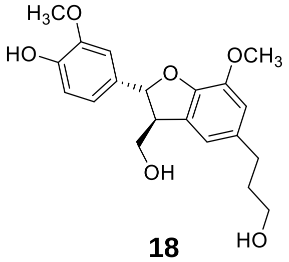

18

(-)-(2*R*,3*S*)-dihydrodehydrodiconiferyl alcohol

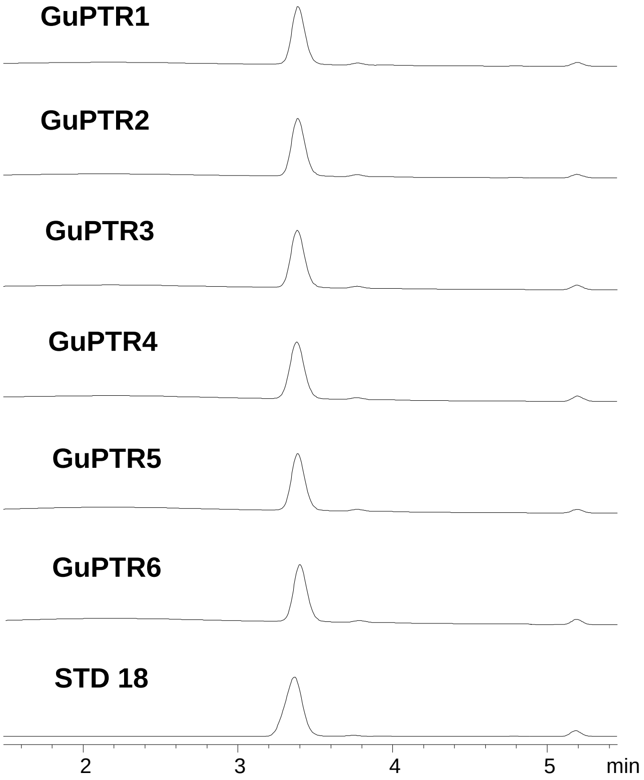

Figure S33. Catalytic activities of GuPTR1–6 toward **18**. Shown are the structure of the substrate and the HPLC/UV chromatograms of the reaction mixtures (λ=280 nm).

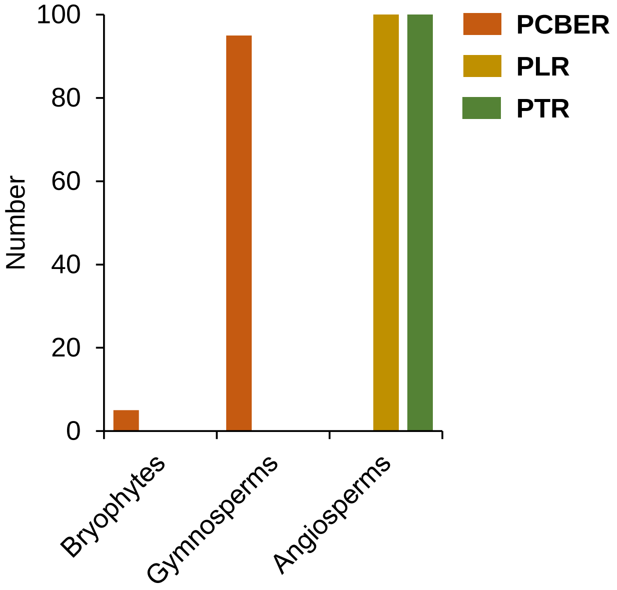

Figure S34. Distribution of PTRs, PLRs, and PCBERs in the plant kingdom.

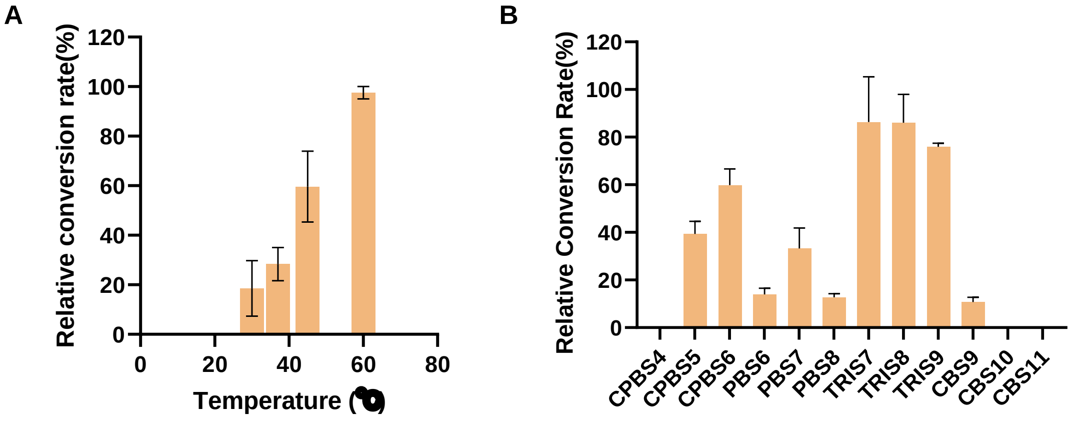

Figure S35. Biochemical properties of N0. (A) Enzymatic activity of N0 at different temperatures (*n*=3). (B) Enzymatic activity of N0 with buffer solutions of different pH values (*n*=3). Compound **17** was used as the substrate.

References

1. Akashi T, Koshimizu S, Aoki T, Ayabe S. Identification of cDNAs encoding pterocarpan reductase involved in isoflavan phytoalexin biosynthesis in *Lotus japonicus* by EST mining. *FEBS Lett* 2006; **580**: 5666–5670.
